# Tyrosinase-mediated synthesis of larvicidal active 1,5-diphenyl pent-4-en-1-one derivatives against *Culex quinquefasciatus* and investigation of their ichthyotoxicity

**DOI:** 10.1038/s41598-021-98281-5

**Published:** 2021-10-20

**Authors:** SathishKumar Chidambaram, Daoud Ali, Saud Alarifi, Raman Gurusamy, SurendraKumar Radhakrishnan, Idhayadhulla Akbar

**Affiliations:** 1 Research Department of Chemistry, Nehru Memorial College (Affiliated to Bharathidasan University), Puthanampatti, Tiruchchirappalli District, Tamil Nadu 621007 India; 2 Department of Zoology, College of Sciences, King Saud University (KSU), P.O. Box 2455, Riyadh, 11451 Saudi Arabia; 3 Department of Life Sciences, Yeungnam University, Gyeongsan, 38541 Gyeongsan-buk South Korea

**Keywords:** Chemical biology, Environmental chemistry, Green chemistry, Chemical synthesis

## Abstract

1,5-diphenylpent-4-en-1-one derivatives were synthesised using the grindstone method with Cu(II)-tyrosinase used as a catalyst. This method showed a high yield under mild reaction conditions. The synthesised compounds were identified by FTIR, ^1^H NMR, ^13^C NMR, mass spectrometry, and elemental analysis. In this study, a total of 17 compounds (**1a–1q**) were synthesised, and their larvicidal and antifeedant activities were evaluated. Compound **1i** (1-(5-oxo-1,5-diphenylpent-1-en-3-yl)-3-(3-phenylallylidene)thiourea) was notably more active (LD_50_: 28.5 µM) against *Culex quinquefasciatus* than permethrin(54.6 µM) and temephos(37.9 µM), whereas compound **1i** at 100 µM caused 0% mortality in *Oreochromis mossambicus* within 24 h in an antifeedant screening, with ichthyotoxicity determined as the death ratio (%) at 24 h. Compounds **1a**, **1e, 1f**, **1j**, and **1k** were found to be highly toxic, whereas **1i** was not toxic in antifeedant screening. Compound **1i** was found to possess a high larvicidal activity against *C. quinquefasciatus* and was non-toxic to non-target aquatic species. Molecular docking studies also supported the finding that **1i** is a potent larvicide with higher binding energy than the control (− 10.0 vs. − 7.6 kcal/mol) in the 3OGN protein. Lead molecules are important for their larvicidal properties and application as insecticides.

## Introduction

In the broadest sense, human beings are part of nature; however, our activity is often understood and interpreted as a category that is unique and separate from the rest of the natural phenomena. It is both the legal and moral obligation of every human to protect planet Earth by undertaking activities that would prevent contamination of our planet and thereby protect it for future generations. For instance, as a scientist in chemical industries or academia, one could focus on protecting nature by employing green chemistry to produce various chemical and pharmaceutical active ingredients. Of the several green chemistry methodologies, the grindstone chemistry technique is a simple practice for the preparation of chemical compounds. Toda et al. developed a range of chemical reactions carried out by simply grinding or triturating the solids together^1^. We will now focus on Mannich reactions, which are a widely studied type of reaction in the organic and medicinal chemistry domains^2^.

Mushroom tyrosinase, which has a dinuclear copper active centre, catalyses the hydroxylation and subsequent oxidation reactions that convert phenol to the related ortho-quinone as well as the oxidation of catechol to quinone^3–8^. Tyrosinase, alongside catechol oxidase^9^ and hemocyanin^10^, belongs to the type 3 copper protein class. The dicopper core of this type-3 copper protein takes three redox forms^3–8^. The active core of the deoxy type [Cu(I)–Cu(I)] contains two cuprous ions, which attach dioxygen to produce the oxy form. Dioxygen bonds as a peroxide ion in the oxy form in the µ-ŋ^2^:ŋ^2^ side-on bridging mode [Cu(II)–O_2_^2−^–Cu(II)]. The met type [Cu(II)–Cu(II)] signifies a condition wherein copper atoms only at the active site have been oxidised but have not been bound by dioxygen. The met type of tyrosinase is an enzymatic form wherein two cupric ions are bridged by one or two tiny ligands, along with water molecules or hydroxide ions, while the enzyme is at rest and acting as a catalyst.

Mannich-type reactions face significant challenges in terms of reaction time, reaction conditions, toxicity, catalyst requirements, and separation and determination of the purity of final product(s). Other challenges include synthetic methodologies such as ultrasound or microwave irradiation, the use of Lewis acids or bases, and the use of solubilizing agents or surfactant-type catalysts^11^. In addition, some of the known green trends in Mannich reactions consist of ball milling without solvents^12^, using ionic liquid mediums^13^, using ionic liquids reinforced with nanoparticles^14^, or applying enzymes under bio-catalytic conditions^15,16^. However, the present study focused on the grindstone green chemistry method in order to overcome the abovementioned challenges in the preparation of Mannich base derivatives.

Mosquitoes are an important transmission vector for several diseases, particularly malaria^17,18^. These types of diseases have economic and social impacts worldwide. Among the mosquito species, *Culex quinquefasciatus* is particularly associated with various vector-spread diseases in several regions. Larvicides are insecticides designed to kill insects during their larval stage. Methoprene is an insect growth controller that prevents larvae from developing significantly beyond the pupa stage by interrupting their growth period. Methoprene is mildly toxic to a variety of crabs, shrimp, lobster, and crayfish and is extremely toxic to a variety of fish and aquatic herbivores; it tends to accumulate in fish tissues^19^. Olfaction plays an important role in many species and is linked to host-seeking, replication, predator recognition, and food detection^20^. Odorant-binding proteins (OBPs) aid signal transduction by transporting odorants to olfactory receptors^21,22^. Some example, consider previous reports, the ligand (5R,6S)-6-acetoxy-5-hexadecanolide^23–25^ was bound to OBP of the *C. quinquefasciatus* mosquito (PDB ID: 3OGN), it is best model for selection 1,5-diphenyl pent-4-en-1-one targets and molecular docking in this study.

**Figure 1:**
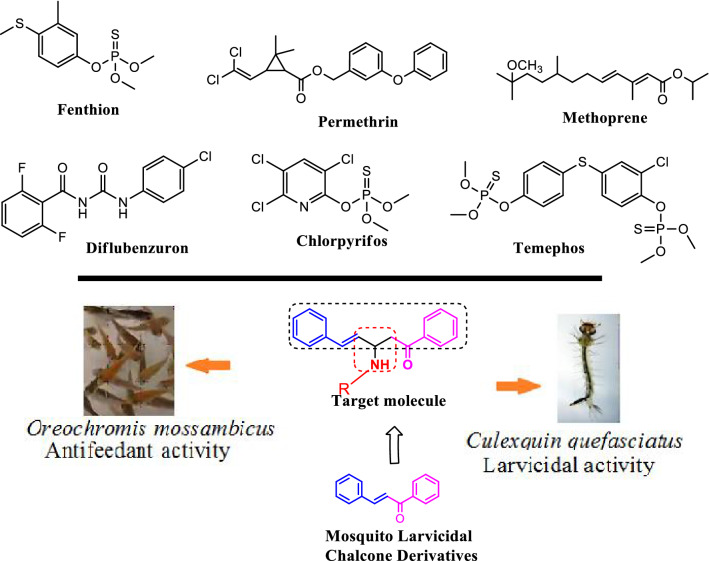
Synthetic marketable insecticides and our target molecule drawn by ChemDraw Ultra 12.0 Suite (PerkinElmer, USA).

The control of mosquitos presents a substantial challenge, and currently inhibitors such as permethrin^26^, organophosphates^27^, fenthion^28,29^, chlorpyrifos^30–32^, temephos^33,34^, diflubenzuron^35^ and methoprene^36^ are used; Fig. 1 details the compositions of these commercial insecticides. However, the use of chemical insecticides pose bigger challenges and various potential environmental problems, such as the widespread development of resistance and disruption of natural biological control systems^37,38^. These problems require overcoming new mosquito larvae inhibitors and improving green methodologies, which can be achieved through Mannich base condensation reactions.

Mannich base synthesis is one of the best tools for green synthesis, in this way preparation of target compound based on cinnamylacetophenone (1,5-diphenylpent-4-en-1-one) comparable to cinnamylphenone (1,3-diphenylprop-2-en-1-one (or) chalcone, Fig. 1), basically chalcone derivatives have mosquito larvicidal properties^39^. Some publications have investigated the environmental study of chalcones^40^ and 1,5-diphenylpent-4-en-1-one (cinnamylacetophenone)^41^. In general, chemical insecticides are the main agents used to reduce populations of vector mosquitoes^42^, even though their accessibility and use are limited by their toxicity to the environment and non-target organisms^43,44^ as well as the resistance of some mosquito species to them.

Chemically modified chalcones have been recently used to control insect populations; for instance, chalcone derivatives are toxic to *Ae. aegypti* first instar larvae and adults^45^ and *Aedes albopictus* larvae^46^. Some furan-chalcones are toxic to *Culex quinquefasciatus* larvae in the fourth stage of development^47^.

The current work was focused on the presence of alkenyl imine/β-amino ketones, particularly imines, which are frequently used in organic synthesis because of their high reactivity and the synthetic utility of the ensuing products^48^. Furthermore, β-amino ketones and their analogues have shown effective medicinal properties^49,50^. So that, current study was to determine novel water-soluble and nontoxic Mannich base 1,5-diphenylpent-4-en-1-one derivatives via grindstone green chemistry methodology that can be used to inhibit the second instar Culex mosquito larvae as a bio-indicator of aquatic pollution.

## Results and discussion

### Chemistry

**Scheme 1:**
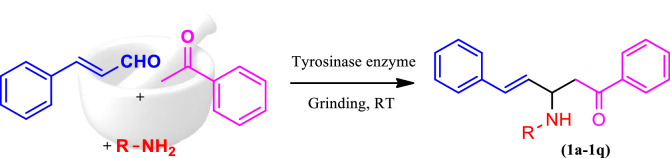
Synthetic route of Mannich base derivative.

**Figure 2:**
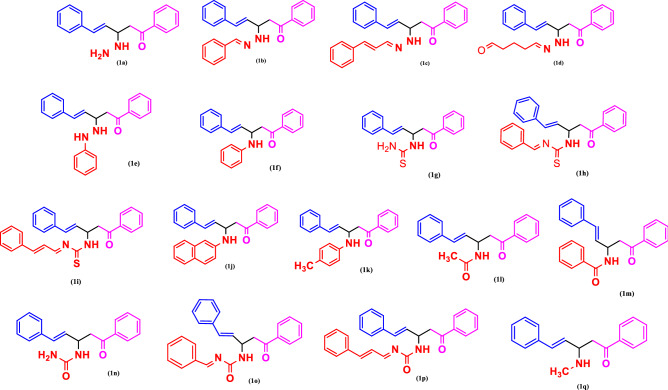
Structures of synthesized Mannich base derivatives (**1a–1q**) drawn by ChemDraw Ultra 12.0 Suite (PerkinElmer, USA).

**Scheme 2:**
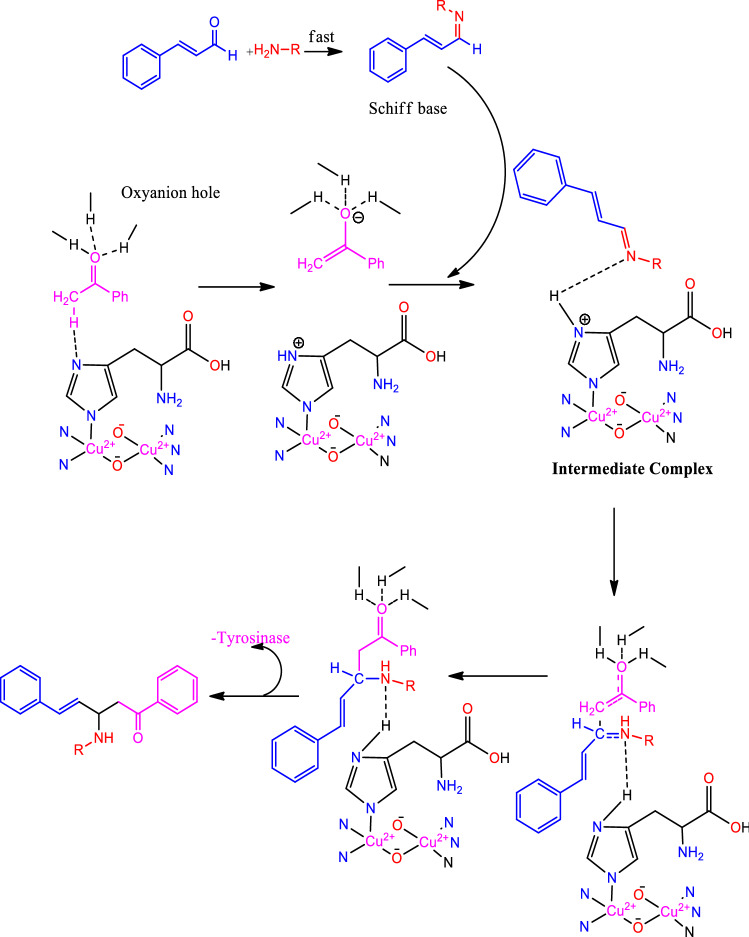
Proposed mechanism of Mannich base derivative formation.

A one-pot multicomponent synthesis of the title compounds was achieved using the grindstone green chemistry method. A mixture of acetophenone, cinnamaldehyde, substituted amine, and a catalytic amount of Cu(II)-tyrosinase enzyme was ground together in a pestle mortar. This was then followed by purification via column chromatography, in order to obtain the title compounds (**1a–1q**). The synthetic route outline is shown in Scheme 1. The chemical structures of synthesized compounds (**1a–1q**) were represented in Fig. 2. The active site in hydrolases is often thought to be responsible for promiscuous catalysis^51^. We suggest a mechanism for the Cu(II)-tyrosinase-catalysed Mannich reaction, outlined in Scheme 2, by combining this perspective with our findings, as mentioned above. First, the aldehyde and amine can easily react to form the Schiff base, and the ketone is simultaneously pre-activated by Cu(II)-tyrosinase to produce the enolate anion. Second, with the aid of the His residue of Cu(II)-tyrosinase, the Schiff base may form an intermediate complex. The Mannich adduct is then freed from the oxyanion hole after a proton is moved from the Schiff base to the enolate anion to create a new carbon–carbon bond. The core steps in this enzymatic mechanism are the formation of the enolate anion and the intermediate complex. Copper-containing materials such as coppertriflate^52^, copperacetate^53^, copperbromide^54^, and copper nanoparticles^55^ play a vital role in Mannich base reactions. The one-pot multicomponent Mannich reaction was catalysed via various enzymes, such as trypsin^56^, lipase^57^, and protease^58^. In the present study, copper containing the Cu(II)-tyrosinase enzyme was used as a catalyst for the synthesis of *N*-Mannich base (**1a–1q**) derivatives.

Some of the previously reported compounds, such as compound **1l**, were reported by β-acetamido ketones from cinnamaldehyde to react with acetophenone at room temperature, with L-proline used as a catalyst, to result in a yield of 75%. Another method was reported previously where *N*-substituted β-amino ketone derivatives had been produced by a one-pot multi-component process using copper(II)-phthalocyanine as a catalyst to result in an yield of 51%, which is comparable to the compound produced in the present work, which showed an 84% yield. Compound **1m** was also reported previously; an imine derived from an α,β-unsaturated aldehyde was also related to the present high binaphthol-derived monophosphoric acids as organocatalysts for enantioselective carbon–carbon bond-forming reactions, thus resulting in a product yield of 81%; an 82% yield was obtained in this study. There is no enzymatic catalysis was involved in the synthesis of compounds **1l** and **1m** in the literature. In our study we utilized Cu(II)-tyrosinase as a catalyst for producing compounds **1l** and **1m** and also the compounds acquired with high yields comparing previous literatures.

The compound **1a** was synthesised using the catalysts trypsin, lipase, protease, CuCl_2_.2H_2_O, and Cu(II)-tyrosinase with yields of 64%, 72%, 68%, 84%, and 92%, respectively. The use of the Cu(II)-tyrosinase enzyme green catalyst, instead of CuCl_2_.2H_2_O, increased the yield of the Mannich derivatives to 92% and reduced the reaction time. The optimisation of the reaction conditions and catalysts is presented in Table 1. The obtained compounds were analysed via FT-IR, ^1^H, and ^13^C NMR spectroscopy. The key assignments of the compounds showed significant bands at 3170.23–3176.54, 2595.45–2599.98, and 1710.68–1716.70 cm^−1^ in the IR spectrum, conforming to the –NH, –C=N, and –C=O groups, respectively. The ^1^H NMR showed signals at δ 8.03–9.70, 3.82–4.81 and 2.40–2.98 ppm, indicating –NH, 4-CH, and –CH_2_ protons, respectively. The ^13^C NMR showed peaks at δ 197.4–197.6, 48.4–59.2, and 48.0–50.6 ppm, which conforms to –C=O, –CH, and –CH_2_ atoms, respectively. Mass spectra and elemental analysis were used to determine the conformation of all these compounds.

**Table 1:** Catalyst optimization for compound **1a.**

Entry	Catalyst	Yield (%)	Time (min)
1	No enzyme	06	30
2	Trypsin from bovine pancreas	64	8
3	Lipase from Candida antarctica	72	12
4	Protease from Streptomyces griseus	68	10
5	CuCl_2_·2H_2_O	84	5
6	Cu(II)-Tyrosinase from mushroom	92	2

“In general, *E*-alkenyl imines are organized from the corresponding *E*-alkenyl aldehydes through imine precursors^59–61^. In this reaction, the carbon–carbon bond formation rate allows the isomerisation of the in situ generated *E*-alkenyl imine from *E*-alkenyl aldehydes with secondary amine and acetophenone, in the presence of 5 mol% of Cu(II)-tyrosinase catalysis to afford the corresponding Mannich adducts (1a-1q) in moderate to good yields with high *E*-selectivity”.

NOE NMR data (see Supplementary Material) clearly confirmed the stereochemistry of the E isomers of compounds **1a**, and **1i**. Thus, based on this study the spectroscopic characteristic downfield shift is observed for this pent-4-en-1-one proton in the *E*-isomer than in the Z-isomer.

### Catalyst recovery studies

**Figure 3:**
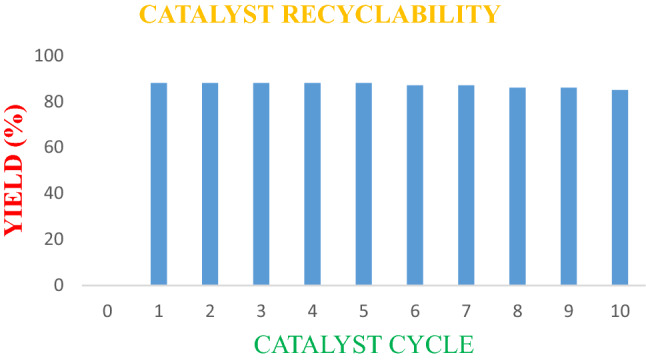
Catalyst recyclability avtivity of Cu(II)-tyrosinase enzyme drawn by Microfoft Office 2019 Suite.

The recovered catalyst was recycled for at least 10 run times with a small defeat in catalytic action (Fig. 3). The decrease in catalytic action perceived through the reinforced catalyst on recycling might be owing to limited loss of basic locates or loss of catalyst surface area during regeneration/reaction. The values are displayed in Table 2.

**Table 2:** Recyclability of Cu(II)-tyrosinase enzyme catalyst.

Entry	Catalyst	Yield (%)
1	1st use	92
2	2nd use	92
3	3rd use	90
4	4th use	90
5	5th use	88
6	6th use	87
7	7th use	87
8	8th use	86
9	9th use	86
10	10th use	85

### Biological activity

A total of 17 compounds (**1a**–**1q**) were tested against second instar *C. quinquefasciatus* larvae, and the toxicity of the title compounds was assessed in the marine fish *Oreochromis mossambicus.* Toxicity was defined as the ratio of deaths (%) at 24 h. Structure–activity relationships showed that the final compounds contained 1,5-diphenylpent-4-en-1-one with different types of amines, thus exerting larvicidal and toxic effects based on the formation of the specific chemical composition.

Compound **1i** showed a higher larvicidal activity than other compounds, with an LD_50_ of 28.5 µM, which was better than that of the controls temephos (LD_50_ of 37.9 µM)^62^ and permethrin (LD_50_ of 54.6 µM). The antifeedant induced 0% mortality even at LD_50_ > 100 µM, which was represented by no toxicity in water.

Compound **1a** induced 80% mortality at 100 µM and its LD_50_ value was 223.0 µM, whereas the antifeedant induced 100% mortality at 100 µM and had a LD_50_ value of 49.5 µM. This suggests that the presence of the hydrazine group may be the reason for the observed antifeedant-induced 100% mortality, as evident from toxicity against *O. mossambicus* fingerlings within 15 min of screening.

Compounds **1f** and **1j** induced a mortality rate of 80% with LD_50_ values of 177.4 µM and 154.9 µM, respectively, in larvicidal screening whereas they induced 100% mortality in antifeedant screening. This suggests that the presence of aniline and naphthalen-2-amine groups may be the reason for the observed biological effects, respectively.

Compounds **1m** and **1n** induced a mortality rate of 80% with LD_50_ values of 159.8 µM and 190.9 µM, respectively, in larvicidal screening whereas they induced 0% mortality in antifeedant screening. This suggests that the presence of the benzamide and urea groups could be the reason for the respective observed biological effects.

Compounds **1d** and **1o** induced 0% mortality at 100 µM in both the larvicidal and antifeedant screening. This suggests that the presence of the 5-hydrazonopentanal and 1-benzylideneurea groups may be the reason for the observed biological effect as they exhibited no active or toxic behaviour.

The above analysis therefore indicates that compound **li** was significantly active in larvicidal screening and displayed low toxicity in antifeedant screening. The percentages of mortality and LD_50_ values are presented in Tables 3 and 4.

**Table 3:** Larvicidal activity of compounds (**1a–1q**).

Compounds	% of Mortality at 25 µM	% of Mortality at 50 µM	% of Mortality at 100 µM	LD_50_ (µM)^a^
**1a**	24.1 ± 0.2	43.2 ± 0.1	80.2 ± 0.2	223.0 ± 0.0
**1b**	11.2 ± 0.2	27.1 ± 0.2	40.1 ± 0.1	282.1 ± 0.0
**1c**	19.3 ± 0.4	26.3 ± 0.4	40.2 ± 0.6	262.8 ± 0.0
**1d**	0 ± 0.0	0 ± 0.0	0 ± 0.0	286.9 ± 0.0
**1e**	33.3 ± 0.1	48.3 ± 0.2	60.4 ± 0.2	193.3 ± 0.3
**1f**	25.0 ± 0.2	44.1 ± 0.2	80.0 ± 0.2	177.4 ± 0.2
**1g**	22.1 ± 0.2	34.2 ± 0.2	40.1 ± 0.3	322.1 ± 0.0
**1h**	34.5 ± 0.2	47.9 ± 0.3	60.1 ± 0.2	165.1 ± 0.2
**1i**	68.2 ± 0.4	88.2 ± 0.6	100 ± 0.0	28.5 ± 0.2
**1j**	26.1 ± 0.2	44.5 ± 0.2	80.4 ± 0.3	154.9 ± 0.2
**1k**	0 ± 0.0	0 ± 0.0	0 ± 0.0	292.8 ± 0.0
**1l**	20.8 ± 0.1	20.8 ± 0.1	20.8 ± 0.1	340.8 ± 0.0
**1m**	29.9 ± 0.3	42.3 ± 0.3	80.9 ± 0.3	159.8 ± 0.2
**1n**	29.9 ± 0.2	43.6 ± 0.2	81.0 ± 0.2	190.9 ± 0.0
**1o**	0 ± 0.0	0 ± 0.0	0 ± 0.0	261.4 ± 0.0
**1p**	40.4 ± 0.1	40.4 ± 0.1	40.4 ± 0.1	244.8 ± 0.0
**1q**	20.4 ± 0.2	20.4 ± 0.2	20.4 ± 0.2	376.8 ± 0.0
**Permethrin**	51.1 ± 1.0	76.3 ± 0.1	100 ± 0.0	54.6 ± 0.0
**Temephos**	56.1 ± 0.2	79.3 ± 0.2	100 ± 0.0	37.9 ± 0.0

Larvicidal activity model is used for the activity assays (second instar *C. quinquefasciatus*), one-day-old larvae were considered as 2nd instar.

^a^Values are mean ± SD (n = 3). Lethal Dose (LD_50_): the LD_50_ is one way to measure the short-term poisoning potential (acute toxicity) of a material.

**Table 4:** Antifeedant activity of compounds (**1a–1q**).

Compounds	% of Mortality at 10 µM	% of Mortality at 25 µM	% of Mortality at 50 µM	% of Mortality at 100 µM	LD_50_ (µM)^a^
**1a**	33.3 ± 0.2	66.2 ± 0.0	88.2 ± 0.0	100 ± 0.0	49.5 ± 0.7
**1b**	20.2 ± 0.3	20.2 ± 0.3	20.2 ± 0.3	20.2 ± 0.3	282.1 ± 0.0
**1c**	0 ± 0.0	0 ± 0.0	0 ± 0.0	0 ± 0.0	262.8 ± 0.0
**1d**	0 ± 0.0	0 ± 0.0	0 ± 0.0	0 ± 0.0	286.9 ± 0.0
**1e**	31.3 ± 0.0	66.1 ± 0.0	82.2 ± 0.0	100 ± 0.0	47.8 ± 0.0
**1f.**	41.2 ± 0.0	51.3 ± 0.0	72.2 ± 0.0	100 ± 0.0	64.4 ± 0.4
**1 g**	–	5.2 ± 0.1	10.3 ± 0.1	20.6 ± 0.2	322.1 ± 0.0
**1 h**	5.3 ± 0.1	20.2 ± 0.1	49.4 ± 0.1	60.4 ± 0.1	131.2 ± 0.8
**1i**	0 ± 0.0	0 ± 0.0	0 ± 0.0	0 ± 0.0	235.5 ± 0.0
**1j**	42.2 ± 0.4	59.2 ± 0.3	88.2 ± 0.0	100 ± 0.0	26.7 ± 0.2
**1 k**	33.1 ± 0.0	67.1 ± 0.74	87.9 ± 0.0	100 ± 0.0	40.4 ± 0.6
**1 l**	–	5.2 ± 0.1	10.3 ± 0.1	20.2 ± 0.1	340.8 ± 0.0
**1 m**	0 ± 0.0	0 ± 0.0	0 ± 0.0	0 ± 0.0	281.3 ± 0.0
**1n**	0 ± 0.0	0 ± 0.0	0 ± 0.0	0 ± 0.0	339.7 ± 0.0
**1o**	0 ± 0.0	0 ± 0.0	0 ± 0.0	0 ± 0.0	261.4 ± 0.0
**1p**	0 ± 0.0	0 ± 0.0	0 ± 0.0	0 ± 0.0	244.8 ± 0.0
**1q**	–	5.2 ± 0.1	10.3 ± 0.1	20.2 ± 1.0	376.8 ± 0.0

Antifeedant activity for the toxicity measurement against marine fish *Oreochromis.*

^a^Values are mean ± SD (n = 3). The LD_50_ is one way to measure the short-term poisoning potential (acute toxicity) of a material.

### Culex quinquefasciatus larval growth regulation

To explore the impact of 1,5-diphenylpent-4-en-1-one formulations on *C. quinquefasciatus* larvae growth, metamorphosis, and production, we exposed the larvae to compound **1i** for 72 h. Table 5 summarizes the effects of compound **1i** impact on larval weight and growth inhibition. When subjected to 10 µM of compound **1i**, the eclosion rate and time of the pupal and adult periods of administered *C. quinquefasciatus* is calculated, and the findings are seen in Table 6. Compound **1i** had a growth-inhibition score of 41.36% and suppressed larval weight development. Furthermore, compound **1i** had little effect on the duration of the adult and pupal periods, but it did result in a 55 percent eclosion rate. Compound **1i** hindered the production and growth of *C. quinquefasciatus* larvae, according to these findings.

**Table 5:** Compound **1i** on the growth of *Culex quinquefasciatus.*

Compound	Weight of larvae (mg)		Weight gain (mg)	Inhibition (%)
	0 h	72 h		
**1i**^**a**^	100.3 ± 1.9	104.1 ± 0.2	3.9 ± 0.9	41.4 ± 2.8
**Control**^**b**^	100.16 ± 0.3	106.7 ± 1.5	6.6 ± 1.4	–

^a^The concentration of 1i was 10 µM.

^b^Control is not containing the compounds.

**Table 6:** Analysis of progress of *Culex quinquefasciatus* growth.

Compound	Duration of pupae (h)	Duration of adult (h)	Rate of eclosion (%)
**1i**^**a**^	68.1 ± 0.64	23.1 ± 1.36	55 ± 1.7
**Control**^**b**^	65.5 ± 1.21	24.2 ± 0.82	80 ± 1.0

^a^The concentration of 1i was 10 µM.

^b^Control is not containing the compounds.

### Docking results

**Figure 4:**
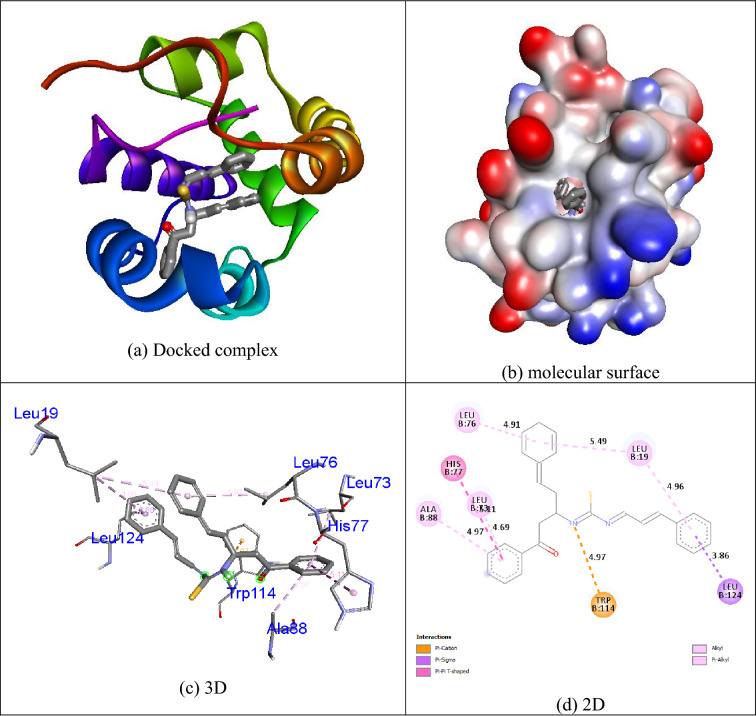
Molecular docking representation of ligand **1i** within the active site of mosquito odorant binding protein (PDB ID: 3OGN). Chemical structures were drawn by ChemDraw Ultra 12.0 Suite (PerkinElmer, USA) and analyzed by the Discovery studio visualizer (BIOVIA Discovery studio 2019 Client).

The Autodock Vina program was used to assess the docking behavior between compounds **1i**, **permethrin** and **temephos** with the mosquito odorant binding protein (PDB ID: 3OGN). Compound **1i** displayed more binding affinity (− 10.0 kcal/mol) than other compounds and **permethrin** (− 9.7 kcal/mol) and **temephos** (− 7.6 kcal/mol) with the mosquito odorant binding protein (PDB ID: 3OGN). Residues of the amino acids Leu19, Leu73, Leu76, His77, Ala78, Trp114, and Leu124 were tangled in hydrophobic connections. The interaction of compound **1i** with mosquito odorant binding protein (PDB ID: 3OGN) is shown in Fig. 4. In the control **permethrin**, residues of the amino acids Leu15, Leu19, Phe59, Leu73, Leu76, His77, Leu80, Ala88, Met89, Gly92, His111, Trp114, Phe123, and Leu124 were tangled in hydrophobic connections.

**Figure 5:**
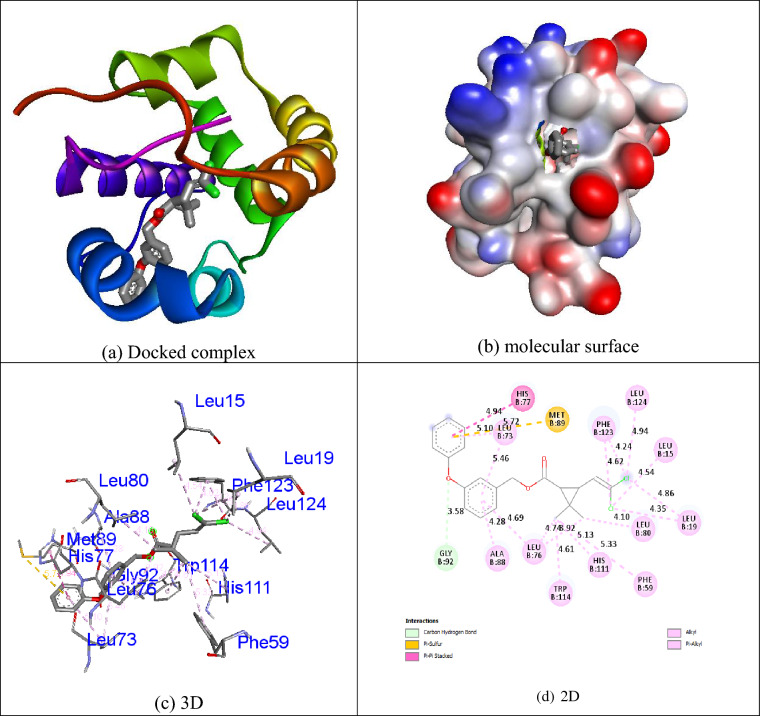
Molecular docking representation of ligand **permethrin** within the active site of mosquito odorant binding protein (PDB ID: 3OGN). Chemical structures were drawn by ChemDraw Ultra 12.0 Suite (PerkinElmer, USA) and analyzed by the Discovery studio visualizer (BIOVIA Discovery studio 2019 Client).

**Figure 6:**
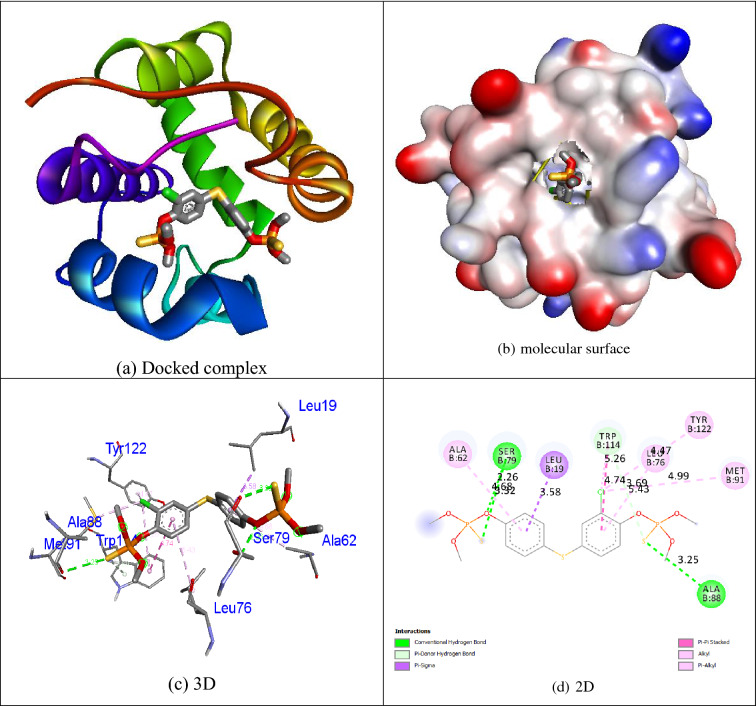
Molecular docking representation of ligand **temephos** within the active site of mosquito odorant binding protein (PDB ID: 3OGN). Chemical structures were drawn by ChemDraw Ultra 12.0 Suite (PerkinElmer, USA) and analyzed by the Discovery studio visualizer (BIOVIA Discovery studio 2019 Client).

The positive control **permethrin** connected in the mosquito odorant binding protein (PDB ID: 3OGN) protein is shown in Fig. 5. The control **temephos** displayed three hydrogen bond interactions with the receptor mosquito odorant binding protein (PDB ID: 3OGN). The amino acid residue Ser79 showed two hydrogen bonds with **temephos**, with the bond lengths of 3.32 and 2.26 Å, and the amino acid residue Ala88 showed one hydrogen bond with **temephos**, with the bond length of 3.25 Å. Residues of the amino acids Leu19, Ala62, Leu76, Met91, Trp114, and Tyr122 were involved in hydrophobic contacts with the receptor. The interaction of the control **temephos** with the mosquito odorant binding protein (PDB ID: 3OGN) protein is shown in Fig. 6. The helix representation of inhibitor molecule docked into the receptor was shown in Figs. 4a, 5a, and 6a. The inhibitor molecule docked into the binding pocket of the receptor was shown in Figs. 4b, 5b, and 6b. The 3D representation of inhibitor molecule docked into the receptor was shown in Figs. 4c, 5c, and 6c. The 2D representation molecule docked with receptor was shown in Figs. 4d, 5d, and 6d. The results show that compound **1i** possesses comparable inhibition abilities relative to the controls **permethrin** and **temephos**. The results are listed in Table 7.

**Table 7:** Molecular docking interaction of compounds (**1a–1q**) and control Temephos, Permethrin.

Compounds	Mosquito odorant-binding protein 3OGN		
	Binding affinity (kcal/mol)	No. of H-bonds	H-bonding residues
**1a**	− 9.0	2	His121, Phe123
**1b**	− 9.7	0	–
**1c**	− 9.0	0	–
**1d**	− 8.8	0	–
**1e**	− 9.7	1	Phe123
**1f**	− 9.6	0	–
**1g**	− 8.3	0	–
**1h**	− 9.3	0	–
**1i**	− 10.0	0	–
**1j**	− 9.8	0	–
**1k**	− 9.8	0	–
**1l**	− 8.9	0	–
**1m**	− 9.8	0	–
**1n**	− 8.8	0	–
**1o**	− 9.5	0	–
**1p**	− 9.2	0	–
**1q**	− 8.3	0	–
**Temephos**	− 7.6	3	Ser79, Ala88
**Permethrin**	− 9.7	0	–

### MD simulation analysis

**Figure 7:**
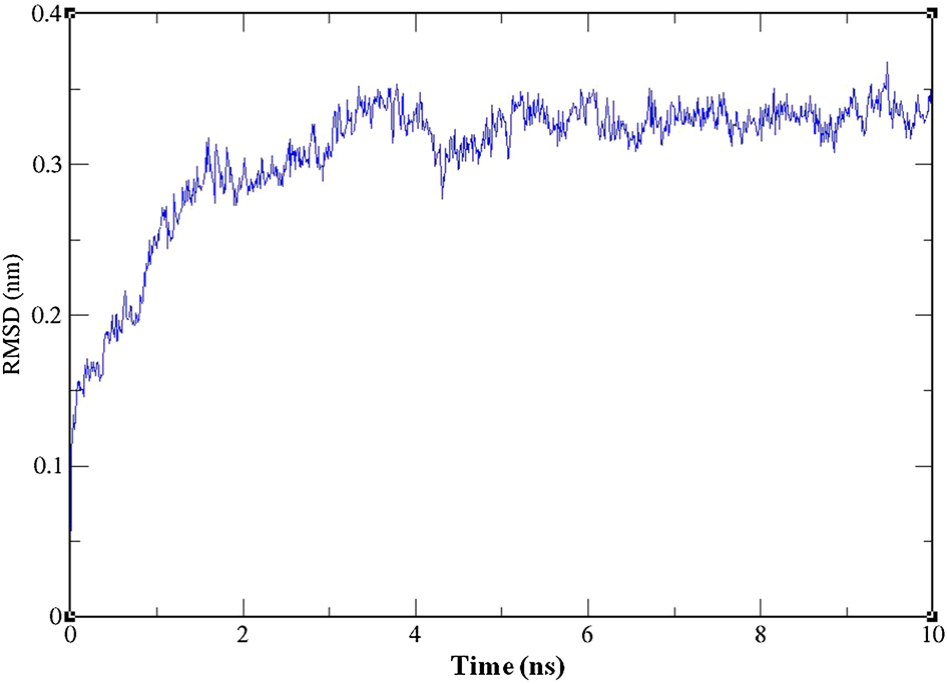
Graphical representation of Time vs. RMSD map for Protein after ligand fit to the protein during molecular dynamics simulation. XMgrace (Version 5.1. 19) tool was used to prepare the graphs (Turner, Land-Margin Research, & Technology, 2005).

The protein–ligand complex structure of ligand **1i** with 3OGN stability was carried out by Molecular Dynamics (MD) simulation method using Gromacs. Root Mean Square Deviation (RMSD) plot is an important to know the stability of the complex structure. From the analysis of values of RMSD plot, the values from 4.5 to 10 ns shows that the structure was stable because Cα backbone of protein was not fluctuated more (Fig. 7).

**Figure 8:**
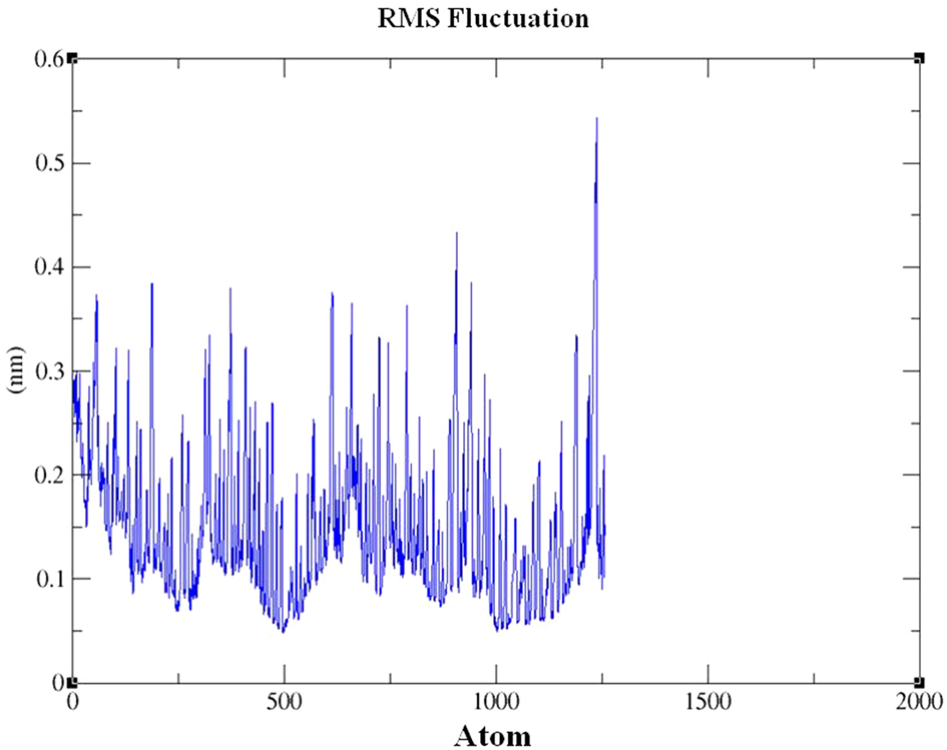
Graphical representation of RMS Fluctuation map during molecular dynamics simulation. XMgrace (Version 5.1. 19) tool was used to prepare the graphs (Turner, Land-Margin Research, & Technology, 2005).

Root Mean Square Fluctuation (RMSF) is an important analysis to characterize the protein residues throughout the simulation time period. From the RMSF analysis, the protein residues other than C terminal were not fluctuated more, especially the residues which were interacted by the ligand Leu 73, Leu 76, His 77, Ala 88, Trp 114 and Leu 124 were within the range of 0.3 nm (Fig. 8).

**Figure 9:**
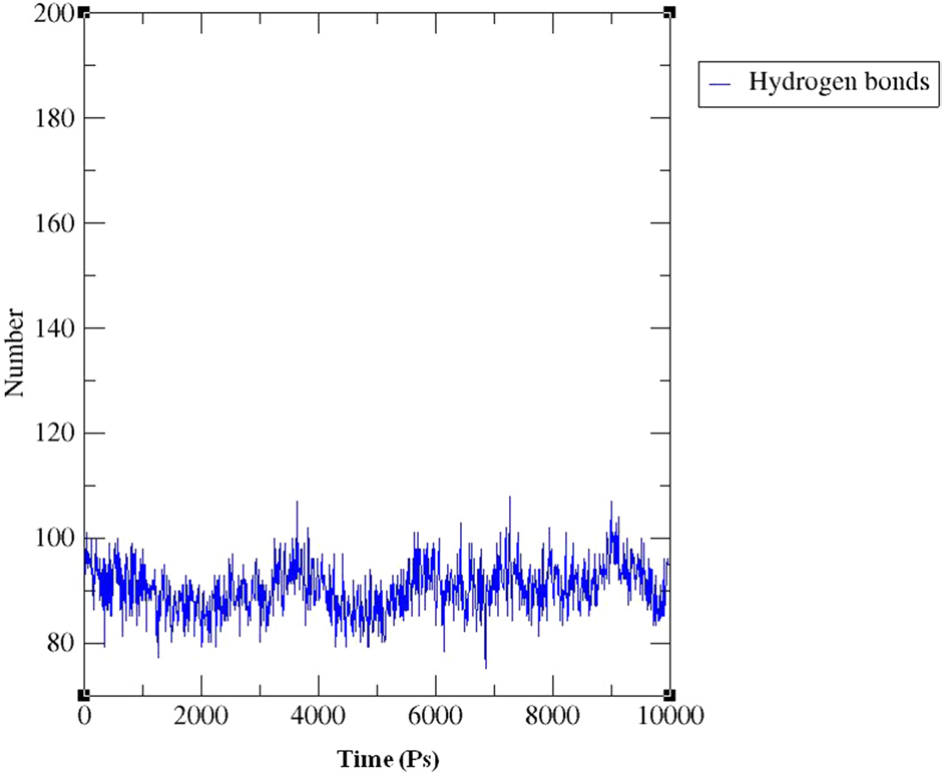
The hydrogen bond interaction between the protein 3OGN and compound **1i.** XMgrace (Version 5.1. 19) tool was used to prepare the graphs (Turner, Land-Margin Research, & Technology, 2005).

The hydrogen bond interaction between the protein 3OGN and ligand **1i** was formed during the period of simulation. 3 hydrogen bonds and pi–pi interaction were formed between the docked complex structures during different nano seconds of simulation system (Fig. 9).

**Figure 10:**
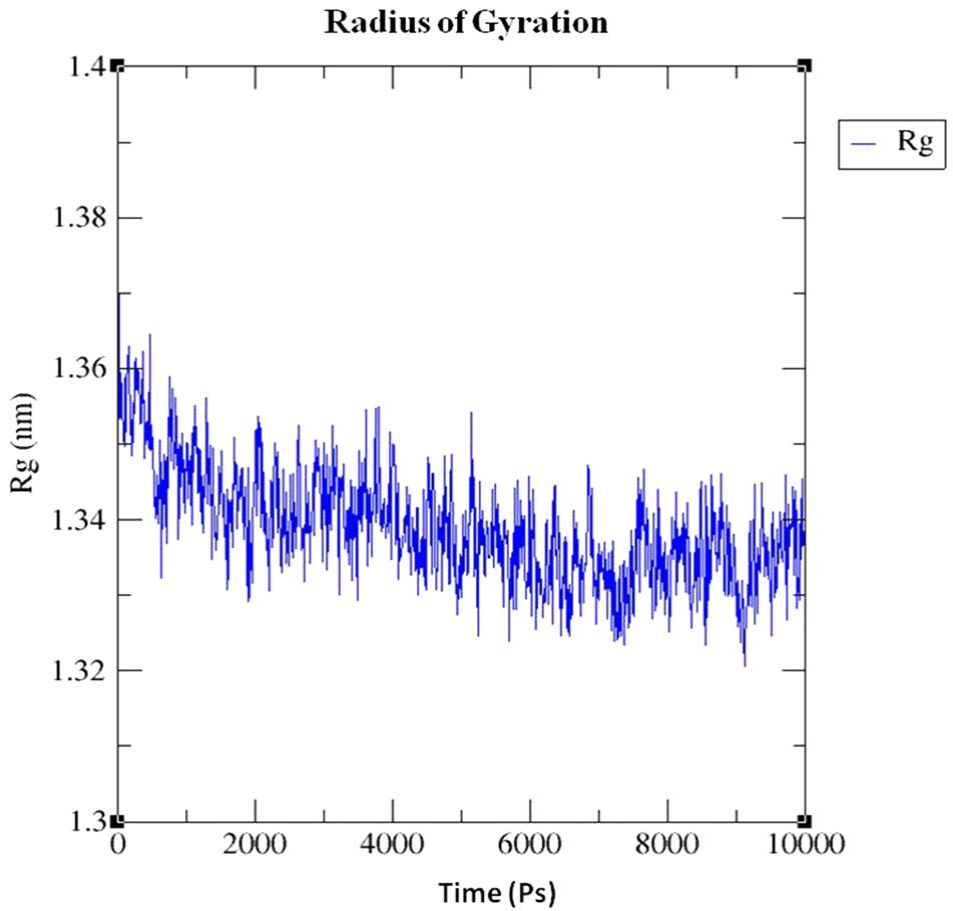
Radius of gyration value of complex structure of protein 3OGN bounded with the compound **1i.** XMgrace (Version 5.1. 19) tool was used to prepare the graphs (Turner, Land-Margin Research, & Technology, 2005).

The radius of gyration value of complex structure of protein 3OGN bounded with the ligand **1i** shows that the ligand causes an alteration of the protein microenvironment. The radius started with 1.36 nm and it is decreased upto 1.33 nm at 6 ns and finally it is increased to 1.34 nm at the 10 ns (Fig. 10).

From this MD simulation analysis, the compound ligand **1i** is stable with the respective of protein and it has good interaction with the important residues of protein. Hence, this compound may suggest to good inhibitor against the 3OGN protein.

## Materials and methods

### Chemistry

Thermo scientific Nicolet iS5 FTIR (4000–400 cm^−1^) was used for analysis of all compounds. Bruker DRX-300 MHz, 75 MHz was used for the analysis of ^1^H and ^13^C NMR spectra. An elemental analyzer (model Vario EL III) was used to analyze elements (C, H, N, and S) percentage (%). Mass spectra were recorded by Perkin Elmer GCMS model Clarus SQ8 (EI).

### General procedure for the synthesis of compounds (1a–1q)

A reaction mixture made up of cinnamaldehyde (0.01 mol, 1.32 mL), acetophenone (0.01 mol, 1.20 mL), substituted amine (0.01 mol) and Cu(II)-tyrosinase enzyme (0.5 g) was mixed in a mortar and ground at RT. Then 2 mL of 50 mM potassium phosphate buffer (pH 6.0) was added and filtered to recover the catalyst. The final filtered solid material was separated using column chromatography (Ethyl acetate4:hexane6). The same method was followed when mixing compounds **1b–1q**.

#### 3-Hydrazinyl-1,5-diphenylpent-4-en-1-one (1a)

White solid; mp: 110–112 °C; Yield: 92%; Water solubility: 0.11 mM/mL; IR(KBr) *ν*: 3171.48, 3065.51, 3041.02, 1715.02, 1624.53 cm^−1^; ^1^H NMR (300 MHz): δ 9.20 (s, 1H), 8.84 (s, 2H, NH_2_), 7.97–7.96 (dd, *J* = 7.33 Hz, *J* = 7.37 Hz, 2H, Ar-ring), 7.63–7.60 (d, *J* = 6.21 Hz, 1H, Ar-ring), 7.53–7.51 (dd, *J* = 7.30 Hz, *J* = 7.34 Hz, 2H, Ar-ring), 7.41–7.37 (dd, *J* = 7.33 Hz, *J* = 7.37 Hz, 2H, Ar-ring), 7.34 (d, *J* = 6.22 Hz, 1H, Ar-ring), 7.21 (dd, *J* = 7.30 Hz, *J* = 7.35 Hz, 2H, Ar-ring), 6.56–6.51 (d, *J* = 6.22 Hz, 1H, CH), 6.19–6.14 (d, *J* = 6.22 Hz, 1H), 3.84–3.80 (m, 1H), 2.94–2.91 (d, *J* = 6.21 Hz, 2H); ^13^C NMR (75 MHz,): 197.4 (1C), 136.7, 133.1, 128.8, 128.6 (6C, Ph ring), 136.4, 128.6, 128.5, 127.9 (6C, Ar ring), 133.4 (1C), 128.4 (1C), 59.2 (1C), 48.0 (1C); EIMS (m/z): 267.15 (M^+^,18%); Anal. Calcd. for C_17_H_18_N_2_O: C, 76.66; H, 6.81; N, 10.52%; found: C, 76.68; H, 6.80; N, 10.51%.

#### 3-(2-Benzylidenehydrazinyl)-1,5-diphenylpent-4-en-1-one (1b)

Greenish solid; mp:145–148 °C; Yield: 86%; Water solubility: 0.06 mM/mL; IR(KBr) *ν*: 3176.51 (NH), 3072.50, 3032.32, 2596.43, 1716.08, 1623.43; ^1^H NMR(300 MHz,): δ 9.21(s,1H), 8.36(s,1H,–CH), 7.97–9.94(dd, *J* = 7.33 Hz, *J* = 7.37 Hz), 7.86–7.81 (dd, *J* = 7.33 Hz, *J* = 7.37 Hz), 7.63–7.60(d, *J* = 6.21 Hz, 1H, Ph), 7.55–7.53(dd, *J* = 7.31 Hz, *J* = 7.34 Hz, 2H), 7.50–7.47(m, 3H, Ar ring), 7.40–7.38(dd, *J* = 7.33 Hz, *J* = 7.37 Hz, 2H, Ar ring), 7.34–7.31(d, *J* = 6.21 Hz, 1H, Ar ring), 7.20–7.17(dd, *J* = 7.31 Hz, *J* = 7.35 Hz, 2H, Ar ring), 6.58–6.54 (d, 1H, *J* = 6.21 Hz, CH), 6.18–6.14(d, *J* = 6.21 Hz, 1H, CH), 3.80–3.76(m, 1H, CH), 2.95–2.92(d, *J* = 6.21 Hz, 2H, CH_2_); ^13^C NMR (75 MHz): 197.6 (1C), 143.3 (1C), 136.6, 133.0, 128.7, 128.5(6C, Ph ring), 136.5, 128.7, 128.6, 128.0(6C, Ar ring), 134.4(1C), 133.7, 131.0, 129.2, 128.8 (6C, Ph ring), 128.5(1C), 55.1(1C), 48.5(1C); EIMS(m/z) 355.18 (M^+^, 26%); Anal. Calcd. for C_24_H_22_N_2_O: C, 81.33; H, 6.26; N, 7.90%; found: C, 81.31; H, 6.27; N, 7.91%.

#### 1,5-Diphenyl-3-(2-(3-phenylallylidene)hydrazinyl)pent-4-en-1-one (1c)

Light green powder; mp: 148–150 °C; Yield: 88%; Water solubility: 0.14 mM/mL**;** IR(KBr) *ν* 3176.50, 3073.51, 3031.30, 2595.45, 1714.08, 1624.40 cm^−1^; ^1^H NMR(300 MHz,): δ 9.26(s, 1H, NH), 7.95–7.91(dd, *J* = 7.33 Hz, *J* = 7.37 Hz), 7.63–7.60–7.58(d, *J* = 6.21 Hz, 1H), 7.57–7.54(dd, *J* = 7.33 Hz, *J* = 7.37 Hz, 2H), 7.53–7.50(dd, *J* = 7.31 Hz, *J* = 7.35 Hz, 2H, Ph), 7.50(s, 1H, CH), 7.40–7.37(dd, *J* = 7.33 Hz, *J* = 7.37 Hz, 4H, Ar ring), 7.36–7.33 (d, *J* = 6.21 Hz, 2H, Ar-ring), 7.24–7.21(dd, *J* = 7.31 Hz, *J* = 7.35 Hz, 2H, Ar ring), 6.54–6.52(d, *J* = 6.21 Hz, 2H, CH), 6.17–6.12(d, *J* = 6.21 Hz, 2H, CH), 3.78–3.74(m, 1H), 2.92–2.89 (d, *J* = 6.21 Hz, 2H); ^13^C NMR (75 MHz,): 197.2 (1C), 137.2 (1C), 136.7, 133.1, 128.8, 128.6, (6C, Ph ring), 136.4, 128.6, 128.5, 127.8 (6C, Ar ring), 135.2, 128.6, 128.5, 127.9(6C, Ph ring), 133.9, 133.7, 128.2, 125.3, 56.2, 48.5; EIMS(m/z): 381.19(M^+^, 28%); Anal. Calcd. for C_26_H_24_N_2_O: C, 82.07; H, 6.36; N, 7.36%; found: C, 82.05; H, 6.37; N, 7.37%.

#### 5-(2-(5-Oxo-1,5-diphenylpent-1-en-3-yl)hydrazono)pentanal (1d)

White powder; mp: 126–129 °C; Yield: 85%; Water solubility: 0.08 mM/mL**;** IR(KBr)*ν* :3176.54, 3073.50, 3031.32, 2595.48, 1714.18, 1624.45; ^1^H NMR (300 MHz,): δ 9.70(s, 1H, CH), 9.24(s, 1H), 7.97–7.94(dd, *J* = 7.33 Hz,*J* = 7.37 Hz, 2H), 7.60–7.57(d, *J* = 6.21 Hz,1H), 7.53–7.50(dd, *J* = 7.31 Hz, *J* = 7.33 Hz, 2H), 7.42–7.37(dd, *J* = 7.33 Hz, *J* = 7.37 Hz, 2H, Ar ring), 7.34–7.31(d, *J* = 6.21 Hz, 1H, Ar ring), 7.21(dd, *J* = 7.31 Hz, *J* = 7.35 Hz, 2H, Ar ring), 6.97(s, 1H, CH), 6.56–6.51(d, *J* = 6.21 Hz, 1H), 6.16–6.13(1H, d, *J* = 6.21 Hz, CH), 3.85–3.82(m, 1H, CH), 2.93–2.88 (d, *J* = 6.21 Hz, 2H), 2.42–2.36(m, 2H), 1.82–1.74(m, 2H), 1.53–1.49 (m, 2H); ^13^C NMR(75 MHz,): 202.2, 197.4, 158.3, 136.7, 133.1, 128.8, 128.6, (6C, Ph ring), 136.4, 128.6, 28.5, 127.9(6C, Ar ring), 134.7, 134.1, 127.9, 56.1, 48.5, 43.3, 25.9(1C); EIMS(m/z): 349.19(M^+^, 24%); Anal.Calcd.for C_22_H_24_N_2_O_2_: C, 75.83; H, 6.94; N, 8.04%; found: C, 75.80; H, 6.96; N, 8.06%.

#### 1,5-Diphenyl-3-(2-phenylhydrazinyl)pent-4-en-1-one (1e)

White powder; mp: 143–145 °C; Yield: 88%; Water solubility: 0.20 mM/mL**;** IR(KBr) *ν*: 3176.52, 3073.50, 3031.28, 1714.10, 1624.38 cm^−1^; ^1^H NMR(300 MHz,): δ 9.22 (s, 1H), 9.16(s, 1H), 7.97(dd, *J* = 7.34 Hz, *J* = 7.38 Hz, 2H, Ph), 7.65(d, *J* = 6.21 Hz, 1H), 7.55–7.53(dd, *J* = 7.31 Hz, *J* = 7.35 Hz, 2H), 7.38–7.34(dd, *J* = 7.33 Hz, *J* = 7.37 Hz, 2H, Ar ring), 7.35–7.32(dd, *J* = 7.31 Hz, *J* = 7.35 Hz, Ph),7.32–7.30(d, *J* = 6.21 Hz, 1H, Ar-ring), 7.21–7.19(dd, *J* = 7.31 Hz, *J* = 7.35 Hz, 2H, Ar-ring), 7.02–6.98(dd, *J* = 7.31 Hz, *J* = 7.35 Hz, 2H, Ph), 6.88–6.86 (d, *J* = 6.21 Hz, 1H, Ar-ring), 6.56–6.54(d, *J* = 6.22 Hz, 1H), 6.17–6.15(d, *J* = 6.21 Hz, 1H), 3.84–3.79(m, 1H), 2.95–2.92(d, *J* = 6.21 Hz, 2H); ^13^C NMR (75 MHz,): 197.4(1C), 136.7, 133.1, 128.8, 128.6, (6C, Ph ring), 136.4, 128.6, 128.5, 127.8 (6C, Ar ring), 151.0, 129.2, 122.8, 113.2 (6C, Ph ring), 134.2, 127.9, 56.6, 48.3; EIMS(m/z): 343.18 (M^+^, 25%); Anal. Calcd. for C_23_H_22_N_2_O: C, 80.67; H, 6.48; N, 8.18%; found: C, 80.65; H, 6.47; N, 8.19%.

#### 1,5-Diphenyl-3-(phenylamino)pent-4-en-1-one (1f)

Yellow powder; mp: 101–103 °C; Yield: 86%; Water solubility: 0.16 mM/mL**;** IR(KBr) *ν***:** 3176.53,3072.50, 3030.28, 1715.10, 1623.38; ^1^H NMR (300 MHz,): δ 9.26(s, 1H, NH), 7.97–7.95(dd, *J* = 7.33 Hz, *J* = 7.37 Hz, 2H), 7.67–7.63(d, *J* = 6.21 Hz, 1H), 7.53–7.51(dd, *J* = 7.31 Hz, *J* = 7.35 Hz, 2H), 7.44–7.41(dd, *J* = 7.33 Hz, *J* = 7.37 Hz, 2H, Ar ring), 7.35–7.30 (d, *J* = 6.21 Hz, 1H, Ar-ring), 7.28–7.23(dd, *J* = 7.31 Hz, *J* = 7.35 Hz, 2H, Ar-ring), 7.25–7.19(dd, *J* = 7.31 Hz, *J* = 7.35 Hz, 2H, Ph), 6.83–6.80(dd, *J* = 7.31 Hz, *J* = 7.35 Hz, 2H, Ph), 6.74–6.71(d, *J* = 6.21 Hz, 1H, Ar ring), 6.56–6.54 (d, *J* = 6.20 Hz, 1H, CH), 6.19–6.17(d, *J* = 6.21 Hz, 1H), 3.84–3.79(m, 1H, -CH), 2.90–2.87(d, *J* = 6.21 Hz); ^13^C NMR(75 MHz,): 197.4(1C), 136.7, 133.1, 128.8, 128.6, (6C, Ph ring), 136.4, 128.6, 128.5, 127.9 (6C, Ar ring), 147.6, 129.5, 120.8, 119.7 (6C, Ph ring), 133.1, 127.7, 57.2, 50.5; EIMS(m/z): 328.17 (M^+^, 25%); Anal. Calcd. for C_23_H_21_NO: C, 84.37; H, 6.46; N, 4.28%; found: C, 84.30; H, 6.49; N, 4.30%.

#### 1-(5-Oxo-1,5-diphenylpent-1-en-3-yl)thiourea (1g)

Green solid; mp: 139–141 °C; Yield: 91%; Water solubility: 0.24 mM/mL**;** IR(KBr) *ν*: 3176.51, 3072.74, 3029.32, 1712.18, 1625.45; ^1^H NMR (300 MHz,) δ 9.22(1H, s, NH), 8.52(s, 2H, NH_2_), 7.97–7.94(dd, *J* = 7.33 Hz, *J* = 7.37 Hz, 2H), 7.63–7.61(d, *J* = 6.21 Hz, 1H, Ph), 7.55–7.50(dd, *J* = 7.31 Hz, *J* = 7.35 Hz, 2H, Ph), 7.40–7.36(dd, *J* = 7.33 Hz, *J* = 7.37 Hz, 2H, Ar ring), 7.33–7.30(1H, d, *J* = 6.21 Hz, Ar ring), 7.23–7.19(dd, 2H, *J* = 7.31 Hz, *J* = 7.35 Hz, Ar ring), 6.56–6.54(d, *J* = 6.22 Hz, 1H), 6.19–6.17(d, *J* = 6.21 Hz, 1H), 3.82–3.79(m, 1H), 2.98–2.96 (d, *J* = 6.20 Hz, 2H); ^13^C NMR(75 MHz,): 197.4(1C), 182.0(1C), 136.7, 133.1, 128.8, 128.6, (6C, Ph ring), 136.4, 128.6, 28.5, 127.9 (6C, Ar ring), 134.2, 128.2, 55.6, 50.6; EI-MS(m/z) 311.12 (M^+^, 19%); Anal. Calcd. for C_18_H_18_N_2_OS: C, 69.65; H, 5.84; N, 9.02%; found: C, 69.68; H, 5.85; N, 9.06%.

#### 1-benzylidene-3-(5-oxo-1,5-diphenylpent-1-en-3-yl)thiourea (1h)

Brown powder; mp: 111–114 °C; Yield: 80%; Water solubility: 0.40 mM/mL; IR(KBr)*ν*: 3175.53, 3070.50, 3032.28, 2597.48, 1714.10,1624.38; ^1^H NMR(300 MHz) δ 9.47(s,1H), 9.26(s,1H), 7.97–7.94(dd, *J* = 7.33 Hz, *J* = 7.37 Hz, 2H, Ar-ring), 7.86–7.84(dd, *J* = 7.31 Hz, *J* = 7.35 Hz, 2H,), 7.63–7.59(d, 1H, *J* = 6.21 Hz, Ar-ring), 7.53–7.51(2H, dd, *J* = 7.31 Hz, *J* = 7.35 Hz Ph), 7.50–7.44(3H, m, Ar-ring), 7.40–7.37(dd, *J* = 7.33 Hz, *J* = 7.37 Hz, 2H, Ar ring), 7.35–7.32(d, *J* = 6.21 Hz, 1H, Ar ring), 7.26–7.24(dd, *J* = 7.31 Hz, *J* = 7.35 Hz, 2H, Ar ring), 6.56–6.54(d, *J* = 6.20 Hz, 1H), 6.19–6.17(d, *J* = 6.21 Hz, 1H), 3.84–3.82(m, 1H), 2.94–2.92(d, *J* = 6.21 Hz, 2H); ^13^C NMR (75 MHz,): 197.4(1C), 182.0(1C), 136.7, 133.1, 128.8, 128.6(6C, Ph ring), 136.4, 128.6, 128.5, 127.9(6C, Ar ring), 135.2, 134.4, 116.1, 20.6 (6C, Ph ring), 134.6, 128.1, 55.6, 50.1, 14.4; EIMS(m/z): 399.15(M^+^,27%); Anal. Calcd. for C_25_H_22_N_2_OS: C, 75.35; H, 5.56; N, 7.03%; found: C, 75.30; H, 5.60; N, 7.04%.

#### 1-(5-Oxo-1,5-diphenylpent-1-en-3-yl)-3-(3-phenylallylidene)thiourea (1i)

Light yellow powder; mp: 276–279 °C; Yield: 87%; Water solubility: 0.10 mM/mL**;** IR(KBr) *ν*: 3174.23, 3069.30, 3031.68, 2598.98, 1715.70, 1626.38; ^1^H NMR (300 MHz,): δ 9.26(s, 1H), 7.98–9.96(dd, *J* = 7.33 Hz, *J* = 7.37 Hz, 2H), 7.65–7.63 (d, *J* = 6.21 Hz, 1H), 7.62–7.59(dd, *J* = 7.31 Hz, *J* = 7.35 Hz, 2H), 7.56–7.54(dd, *J* = 7.31 Hz, *J* = 7.35 Hz, 2H), 7.52(s, 1H), 7.42–7.39 (dd, *J* = 7.33 Hz, *J* = 7.37 Hz, 4H,Ar ring), 7.31–7.27(d, *J* = 6.21 Hz, 2H, Ar ring), 7.26–7.24(dd, *J* = 7.31 Hz, *J* = 7.35 Hz, 2H, Ar ring), 7.22–7.18(d, *J* = 6.21 Hz, 1H, CH), 6.81–6.79(d, *J* = 6.23 Hz, 1H), 6.56–6.52(d, *J* = 6.21 Hz, 1H), 6.19–6.17(d, *J* = 6.21 Hz, 1H, CH), 3.84–3.79(m, 1H), 2.94–6.92(d, *J* = 6.21 Hz, 2H); ^13^C NMR(75 MHz,): 197.4 (1C), 189.3(1C), 163.7, 136.7, 133.1, 128.8, 128.6, (6C, Ph ring), 136.4, 128.6, 128.5, 127.9(6C, Ar ring), 135.2, 134.4, 116.1, 20.6(6C, Ph ring), 134.6, 132.9, 128.3, 119.9, 55.9, 50.6; EIMS(m/z) 425.16 (M^+^, 30%); Anal. Calcd. for C_27_H_24_N_2_OS: C, 76.38; H, 5.70; N, 6.60%; found: C, 76.30; H, 5.74; N, 6.62%.

#### 3-(Naphthalen-2-ylamino)-1,5-diphenylpent-4-en-1-one (1j)

Dark yellow colour; mp: 101–104 °C; Yield: 88%; Water solubility: 0.32 mM/mL; IR(KBr) *ν*: 3174.63, 3069.70, 3031.48, 1715.50, 1626.48 cm^−1^; ^1^H NMR (300 MHz,): δ 9.26(s, 1H, NH), 7.97–7.94 (dd, *J* = 7.33 Hz, *J* = 7.37 Hz, 2H, Ph), 7.88–7.84 (d, *J* = 6.21 Hz, 1H, Napthyl), 7.83–7.81(d, *J* = 6.21 Hz, 1H, Napthyl), 7.77–7.74(d, *J* = 6.21 Hz, 1H, Napthyl), 7.49–7.45 (d, *J* = 6.21 Hz, 1H, Napthyl), 7.45–7.41 (d, *J* = 6.21 Hz, 1H, Napthyl), 7.50 -7.48(dd, *J* = 7.31 Hz, *J* = 7.35 Hz, 2H, Naphthyl), 7.63–7.59(d, *J* = 6.23 Hz, 1H, Ph), 7.53–750(dd, *J* = 7.31 Hz, *J* = 7.35 Hz, 2H, Ar-ring), 7.42–7.40 (dd, *J* = 7.33 Hz, *J* = 7.37 Hz, 2H, Ar ring), 7.35–7.33(d, *J* = 6.21 Hz, 1H, Ar-ring), 7.25–7.21(dd, *J* = 7.31 Hz, *J* = 7.35 Hz, 2H, Ar, ring), 6.56 -6.54(d, *J* = 6.21 Hz, 1H, CH), 6.19–6.17(d, *J* = 6.21 Hz, 1H), 3.84–3.81(m, 1H), 2.90–2.87(d, *J* = 6.21 Hz, 2H); ^13^C NMR (75 MHz,): 197.4(1C), 136.7, 133.1, 128.8, 128.6(6C, Ph ring), 136.4, 128.6, 128.5, 127.9(6C, Ar ring), 146.0, 133.7, 129.0, 126.8, 126.5, 125.3, 124.6, 121.4, 118.1, 104.5(10C, Naphthyl ring), 134.4, 128.1, 57.2, 50.5; EI-MS(m/z) 378.18 (M^+^, 29%); Anal. Calcd. for C_27_H_23_NO: C, 85.91; H, 6.14; N, 3.71%; found: C, 85.90; H, 6.10; N, 3.76%.

#### 1,5-Diphenyl-3-(p-tolylamino)pent-4-en-1-one (1k)

White powder; mp: 72–74 °C; Yield: 85%; Water solubility: 0.26 mM/mL; IR(KBr) *ν*: 3173.23, 3068.30, 3030.68, 1714.70, 1625.38; ^1^H NMR (300 MHz): δ 9.28(s, 1H), 7.50(s, 1H, –CH), 7.97–7.96(dd, *J* = 7.35 Hz, *J* = 7.39 Hz, 2H, Ph), 7.64(d, *J* = 6.21 Hz, 1H), 7.53(dd, *J* = 7.31 Hz, *J* = 7.34 Hz, 2H), 7.39(dd, *J* = 7.33 Hz, *J* = 7.37 Hz, 4H, Ar-ring), 7.33–7.31(d, *J* = 6.21 Hz, 2H, Ar-ring), 7.24–7.20(dd, *J* = 7.31 Hz, *J* = 7.35 Hz, 2H, Ar-ring), 7.22–7.18(d, *J* = 6.21 Hz,1H), 7.01–6.98 (dd, *J* = 7.31 Hz, *J* = 7.35 Hz, 1H, Ph), 6.85–6.84(1H, d, *J* = 6.21 Hz), 2.90–2.87 (d, *J* = 6.21 Hz, 2H), 2.34(s, 3H); ^13^C NMR (75 MHz,): 197.4(1C), 136.7, 133.1, 128.8, 128.6(6C, Ph ring), 136.4, 128.6, 128.5, 127.9 (6C, Ar ring), 144.6, 129.8, 129.6, 113.4(6C, 4-CH_3_-Ph ring), 134.5, 128.6, 55.2, 50.6, 21.3; EIMS(m/z) 342.18(M^+^, 26%); Anal. Calcd. for C_24_H_23_NO: C,84.42; H, 6.79; N, 4.10%; found: C, 84.30; H, 6.89; N, 4.12%.

#### N-(5-oxo-1,5-diphenylpent-1-en-3-yl)acetamide (1l)

Pale yellow powder; mp: 122–124 °C; Yield: 84%; Water solubility: 0.15 mM/mL; IR(KBr)*ν*: 3170.23, 3065.30, 3027.68, 1711.70, 1622.38; ^1^H NMR (300 MHz,): δ 8.05(s, 1H, NH), 7.95–7.92 (dd, *J* = 7.31 Hz, *J* = 7.36 Hz, 2H, Ph), 7.65–7.64(d, *J* = 6.21 Hz, 1H), 7.54–7.50(dd, *J* = 7.31 Hz, *J* = 7.35 Hz, 2H, Ar-ring), 7.38–7.34(dd, *J* = 7.31 Hz, *J* = 7.35 Hz, 1H, Ar ring), 7.31–7.28(d, *J* = 6.21 Hz, 2H, Ar-ring), 7.25–7.21(dd, *J* = 7.31 Hz, *J* = 7.35 Hz, 2H, Ar ring), 6.56–6.53(d, *J* = 6.21 Hz, 1H, CH), 6.17–6.15(d, *J* = 6.21 Hz, 1H), 4.81–4.78 (m, 1H), 2.94–2.91(d, *J* = 6.21 Hz,2H), 1.84 (s, 3H); ^13^C NMR (75 MHz,): 197.4(1C), 170.7(1C), 136.7, 133.1, 128.8, 128.6(6C, Ph ring), 136.4, 128.6, 128.5, 127.9(6C, Ar ring), 134.1, 127.9, 48.4, 50.4, 23.7; EIMS(m/z) 294.14 (M^+^, 20%); Anal. Calcd. for C_19_H_19_NO_2_: C, 77.79; H, 6.53; N, 4.77%; found: C, 77.80; H, 6.51; N, 4.75%.

#### N-(5-oxo-1,5-diphenylpent-1-en-3-yl)benzamide (1m)

Brown powder; mp: 205–208 °C; Yield: 82%; Water solubility: 0.34 mM/mL; IR(KBr) *ν*: 3172.21, 3063.28, 3025.66, 1710.68, 1620.36; ^1^H NMR (300 MHz): δ 8.41(s, 1H, NH), 8.03–7.96(dd, *J* = 7.33 Hz, *J* = 7.37 Hz, 2H, Ar-ring), 7.97(dd, *J* = 7.32 Hz, *J* = 7.34 Hz, 2H), 7.70–7.67(1H, d, *J* = 6.21 Hz, Ar ring), 7.63–7.60(3H, m, Phenyl), 7.53–7.50(dd, *J* = 7.31 Hz, *J* = 7.33 Hz, 2H), 7.42–7.38 (dd, *J* = 7.33 Hz, *J* = 7.37 Hz, 4H, Ar ring), 7.33–7.30 (1H, d, *J* = 6.21 Hz, Ph), 6.51–6.49(d, *J* = 6.21 Hz, 1H), 6.19–6.17(d, *J* = 6.21 Hz, 1H), 4.81–4.78(1H, m,–CH), 2.98–2.95(d, *J* = 6.21 Hz, 2H); ^13^C NMR (75 MHz,): 197.4(1C), 167.5(1C), 136.7, 133.1, 128.8, 128.6, (6C, Ph ring), 136.4, 128.6, 128.5, 127.9 (6C, Ar ring), 134.2, 132.1, 128.8, 127.5 (6C, Ph ring), 135.1, 127.9, 49.2, 50.4; EIMS(m/z): 356.16 (M^+^, 26%); Anal. Calcd. for C_24_H_21_NO_2_: C, 81.10; H, 5.96; N, 3.94%; found: C, 80.10; H, 5.92; N, 4.04%.

#### 1-(5-Oxo-1,5-diphenylpent-1-en-3-yl)urea (1n)

Pale green powder; mp: 260- 262 °C**;** Yield: 82%; Water solubility: 0.40 mM/mL IR (KBr) *ν*: 3173.21, 3064.28, 3026.66, 1711.68, 1621.36; ^1^H NMR (300 MHz,): δ 9.22(s, 1H, NH), 8.83(s, 2H, NH_2_), 7.97–7.94 (dd, *J* = 7.33 Hz, *J* = 7.37 Hz, 2H), 7.64–7.59 (m, 1H, Phenyl), 7.55–7.53(dd, *J* = 7.31 Hz, *J* = 7.35 Hz, 2H), 7.40–7.37(dd, *J* = 7.35 Hz, *J* = 7.33 Hz, 1H, Ar ring), 7.34–7.31(d, *J* = 6.21 Hz, 2H, Ar ring), 7.23–7.18(dd, *J* = 7.31 Hz, *J* = 7.35 Hz, 2H, Ar ring), 6.56–6.54(d, *J* = 6.20 Hz, 1H), 6.17–6.15(d, *J* = 6.21 Hz, 1H), 4.81–4.78 (m, 1H), 2.94–2.91 (d, *J* = 6.21 Hz, 2H); ^13^C NMR(75 MHz,): 197.4 (1C), 162.7 (1C), 136.7, 133.1, 128.8, 128.6, (6C, Ph ring), 136.4, 128.6, 128.5, 127.9(6C, Ar ring), 133.9, 128.9, 50.5, 49.9; EIMS(m/z): 295.14 (M^+^, 19%); Anal. Calcd. for C_18_H_18_N_2_O_2_: C, 73.45; H, 6.16; N, 9.52%; found: C, 73.40; H, 6.17; N, 9.54%.

#### 1-Benzylidene-3-(5-oxo-1,5-diphenylpent-1-en-3-yl)urea (1o)

Green solid; mp: 132–135 °C; Yield: 80%; Water solubility: 0.18 mM/mL; IR(KBr) *νJ*: 3174.23, 3069.30, 3031.68, 2598.98, 1715.70, 1626.38; ^1^H NMR (300 MHz) δ 9.48(s, 1H), 8.06 (s, 1H), 7.97(dd, *J* = 7.33 Hz,  = 7.37 Hz, 2H), 7.85–7.53(dd, *J* = 7.31 Hz, *J* = 7.35 Hz, 2H, Phenyl), 7.60–7.57 (dd, *J* = 7.31 Hz, *J* = 7.35 Hz, 1H), 7.63–7.60 (d, *J* = 6.21 Hz, 1H, Phenyl), 7.55–7.52(dd, *J* = 7.31 Hz, *J* = 7.35 Hz, 2H), 7.52 (m, 2H, Ph), 7.40–7.37(dd, *J* = 7.35 Hz, *J* = 7.38 Hz, 1H, Ar ring), 7.35–7.31(d, *J* = 6.21 Hz, 2H, Ar ring), 7.27–7.23(dd, *J* = 7.31 Hz, *J* = 7.35 Hz, 2H, Ar ring), 6.56–6.54 (d, *J* = 6.21 Hz, 1H), 6.19–6.16(d, *J* = 6.22 Hz, 1H), 4.81–4.79 (m, 1H), 2.94 (d, *J* = 6.21 Hz, 2H); ^13^C NMR (75 MHz): 197.4(1C), 164.5 (1C), 163.7 (1C), 136.7, 133.1, 128.8, 128.6(6C, Ph ring), 136.4, 128.6, 128.5, 127.9 (6C, Ar ring), 133.7, 131.0, 129.2, 128.8(6C, Ph ring), 133.8, 127.7, 50.8, 49.9; EIMS(m/z): 383.17 (M^+^, 28%); Anal. Calcd. for C_25_H_22_N_2_O_2_: C, 78.51; H, 5.80; N, 7.32%; found: C, 78.50; H, 5.82; N, 7.31%.

#### 1-(5-Oxo-1,5-diphenylpent-1-en-3-yl)-3-(3-phenylallylidene)urea (1p)

White greenish powder; mp: 145–148 °C; Yield: 89%; Water solubility: 0.52 mM/mL**;** IR(KBr) *ν***:** 3175.23,3070.30,3032.68, 2599.98, 1716.70, 1627.38; ^1^H NMR (300 MHz,): δ 8.04(s, 1H), 7.50(s, 1H), 7.96–7.93(dd, *J* = 7.33 Hz, *J* = 7.37 Hz, 2H, Ar-ring), 7.60–7.54(dd, *J* = 7.31 Hz, *J* = 7.35 Hz, 2H, Ar-ring), 7.64–7.59(d, *J* = 6.21 Hz,1H, Ph), 7.54–7.51(dd, *J* = 7.31 Hz, *J* = 7.35 Hz, 2H), 7.42–7.39(dd, *J* = 7.33 Hz, *J* = 7.37 Hz, 4H, Ar-ring), 7.32–7.28(d, *J* = 6.21 Hz, 2H, Ar ring), 7.28–7.25 (dd, *J* = 7.31 Hz, *J* = 7.35 Hz, 2H, Ar ring), 7.24–7.21(d, *J* = 6.21 Hz, 1H, CH), 6.85–6.83 (d, *J* = 6.21 Hz, 1H), 6.54–6.51 (d, *J* = 6.21 Hz, 1H, CH), 6.17–6.13(d, *J* = 6.21 Hz, 1H, CH), 4.81–4.78 (m, 1H), 2.94–2.92 (d, *J* = 6.21 Hz, 2H); ^13^C NMR (75 MHz,): 197.4(1C), 164.5(1C), 163.7 (1C), 136.7, 133.1, 128.8, 128.6(6C, Ph ring), 136.4, 128.6, 128.5, 127.9(6C, Ar ring), 135.2, 134.4, 116.1, 20.6(6C, Ph ring), 134.1, 133.5, 128.5, 119.9, 50.8, 49.9; EI-MS: 409.19 (M^+^, 29%); Elemental analysis**:** Anal. Calcd. for C_27_H_24_N_2_O_2_: C, 79.39; H, 5.92; N, 6.86%; found: C, 79.30; H, 5.96; N, 6.91%.

#### 3-(Methylamino)-1,5-diphenylpent-4-en-1-one (1q)

Light yellow powder; mp: 84–88 °C; Yield: 86%; Water solubility: 0.46 mM/mL; IR(KBr) *ν*: 3173.21, 3064.28, 3026.66, 1711.68, 1621.36; ^1^H NMR(300 MHz) δ 9.26(s, 1H, NH), 7.97–7.94(dd, *J* = 7.31 Hz, *J* = 7.36 Hz, 2H, Ph), 7.68–7.62 (m,1H,Ar-ring), 7.51–7.48(dd, *J* = 7.31 Hz, *J* = 7.35 Hz, 2H), 7.41–7.37(dd, *J* = 7.33 Hz, *J* = 7.37 Hz, 2H, Ar-ring), 7.31–7.29(d, *J* = 6.21 Hz, 2H, Ar ring), 7.25–7.19(dd, *J* = 7.31 Hz, *J* = 7.35 Hz, 1H, Ar ring), 6.56–6.54 (d, *J* = 6.21 Hz, 1H, CH), 6.19–6.17(d, *J* = 6.20 Hz, 1H), 3.84–3.81 (m, 1H, –CH), 3.36(s, 3H), 2.79–2.77(d, *J* = 6.20 Hz, 2H); ^13^C NMR (75 MHz): 197.4(1C), 136.7, 133.1, 128.8, 128.6(6C, Ph ring), 136.4, 128.6,128.5, 127.9(6C, Ar-ring), 134.6 (1C), 127.5, 57.1, 50.3, 23.1; EIMS(m/z): 266.15 (M^+^, 19%); Anal. Calcd. for C_18_H_19_NO: C, 81.47; H, 7.22; N, 5.28%; found: C, 81.40; H, 7.25; N, 5.32%.

### Biological activities

#### Larvicidal activity

Larvicidal activity assessed to control the breed of mosquitoes at their larval stage by using chemical compounds as larvicides. Test compounds were deviated in various concentrations of 10, 25, 50 and 100 µM according to a method described previously^16^. Mortality caused by the compounds was assessed as ratios (%) of the numbers of dead vs. live larvae. The LD_50_ values were calculated using probit analysis.

#### Antifeedant activity

Antifeedant activity was evaluated to study the effect of larvicides against non-target aquatic species. The antifeedant activity was screened via 10, 25, 50 and 100 µM concentrations of the tested samples and evaluated for marine fingerlings (*O. mossambicus*). Mortality caused by the compounds was assessed as ratios (%) of the numbers of dead vs. live fingerlings. Table 4 summarizes the results. The method followed was described previously^16^.

#### Larval growth inhibition and regulation

The regulation and inhibition of larval growth in *C. quinquefasciatus* by compound **1i** (10 µM) were analysed via the water-immersion method^63^.

### Molecular docking

#### Preparation of ligands

The ligand molecules (**1a–1q**) were drawn via Chemdraw 12.0 and energy was minimized by using the MM2 force field in Chem3Dpro software. The ligand molecules were then saved in Protein Data Bank (PDB) format and further used for molecular docking studies.

#### Preparation of receptor

The 3D crystal structure of mosquito odorant binding protein (PDB ID: 3OGN) was downloaded from Protein Data Bank. The water molecules and inbound co-crystallized ligands were removed from the receptor using the Discovery Studio 2019 program. The receptor was energy minimized via the SWISS PDB Viewer program. The receptor was then used for molecular docking evaluation.

#### Identification of binding pocket

The binding pocket of the target protein was recognized by using inbound co-crystallized ligands via the Discovery Studio 2019 Program. Residues of the amino acids Tyr10, Leu15, Leu19, Leu73, Leu80, Met84, Ile87, Ala88, Met91, His111, Trp114, His121, and Phe123 were situated in the binding pocket.

#### Docking

The interaction of binding modes between compounds **1a–1q**, **permethrin, temephos** (see Supplementary Material) and the mosquito odorant binding protein was assessed using molecular docking studies via Autodock vina 1.1.2. software^64^. The selection of docking grid box was based on the active amino acid residues situated on the binding pocket. The search grid of the 3OGN protein was stable with the dimensions sizes x: 22, y: 20, and z: 22 with center_x: 18.681, y: 49.66, and z: 11.409, with a spacing of 1.0 Å^65^. The value of exhaustiveness was set to 8 and the interactions were visually examined using the Pymol and Discovery studio 2019 programs.

#### Molecular dynamics simulations

Gromacs 2020.1 version was used to carry out the Molecular dynamics simulation for docked complex structure of ligand **1i** with protein 3OGN to understand the stability of the docked complexes. Ligand topology was generated using PRODRG server and it is combined with protein topology for making complex topology, the system was generated using force field GROMOS 43a1, solvated using a single point charge (SPC) water model. The system was framed by cubic box with a distance of 2 nm from the box to the surface of the protein.

The necessary ions were further added in order to neutralize the systems. The docked complex energy was minimized by energy minimization process using steepest descent algorithm, for each simulation, 50,000 steps were used for energy minimization. The LINCS algorithm was used to constrained the bond lengths and the electrostatics computed by PME method. NVT and NPT ensembles were used to equilibrating the systems for each 100 ps. The V-rescale thermostat was used for equilibration with a reference temperature of 300 K. Finally, the production MD run was approved for 10 ns with a time-step of 2 fs. Docked complex structure coordinates were hoarded every 10 ps and used for further analysis. The result was analysed through the RMSD, RMSF, gyration, hydrogen bonds plots and Xmgrace software was used for plotting graphs.

### Statistical analysis

The LD_50_ values was calculated based on at least three independent assessments and the standard deviations (SD) were calculated using Microsoft Excel.

## Conclusions

In this study, we identified the most effective and easily prepared active larvicidal Mannich base synthesis derivatives using the grindstone method using Cu(II)-tyrosinase as a catalyst, which is economical and leads to good coating and high yield. These compounds were investigated for their use as larvicides against *Culex quinquefasciatus* and for their toxicity against non-target aquatic species through ichthyotoxic activity. A total of 17 compounds were screened, and compound **1i** was found to be the most active (LD_50_ = 12.09 µM) against *Culex quinquefasciatus* compared to **Permethrin** (LD_50_ = 54.6 µM). The compound **1i** was highly active compared to **Permethrin** > 10 differences compared with standard permethrin and also compound **1i** induced 0% mortality within 24 h against *Oreochromis mossambicus* in an antifeedant screening. Molecular docking was carried out with all compounds **1a–1q** and the controls **temephos** and **permethrin** against the 3OGN protein, and the resulting docking score was the best for compound **li**. In conclusion, our results indicate that compound **li** is the most effective insecticide and that the compounds outlined in this paper may serve as a prospective foundation for emerging ecologically significant bioactive compounds as well as eco-friendly pesticides and biopharmaceuticals.

## Supplementary Information

Supplementary Information.
