# Supplementary material for: Tyrosinase-mediated synthesis of larvicidal active 1,5-diphenyl pent-4-en-1-one derivatives against Culex quinquefasciatus and investigation of their ichthyotoxicity
Source: Sci Rep. 2021 Oct 20;11:20730. doi: 10.1038/s41598-021-98281-5 (PMC8528871; doi:10.1038/s41598-021-98281-5)
Supplement: Supplementary file 1 — Supplementary Information. [file 41598_2021_98281_MOESM1_ESM.pdf]

Tyrosinase-mediated synthesis of larvicidal active 1,5-diphenyl pent-4-en-1-one derivatives against *Culex quinquefasciatus* and investigation of their ichthyotoxicity

Chidambaram SathishKumar[1], Daoud ali[2], Saud alarifi[2], Radhakrishnan SurendraKumar[1], and Akbar Idhayadhulla[1*]

[1] Research Department of Chemistry, Nehru Memorial College (Affiliated to Bharathidasan University), Puthanampatti -621007, Tiruchirappalli District, Tamil Nadu, India.
[2] Department of Zoology, College of Sciences, King Saud University (KSU), Riyadh, Saudi Arabia.
* Correspondence: a.idhayadhulla@gmail.com, idhayadhulla@nmc.ac.in

S.No.	Contents	Page no.
1	^{1}H , and ^{13}C NMR spectrum of Compound **(1a-1q)**	1 - 17
2	**Table S1.** Molecular docking interaction of compounds (**1a-1q**) and controls **Temephos**, **Permethrin**	18- 24

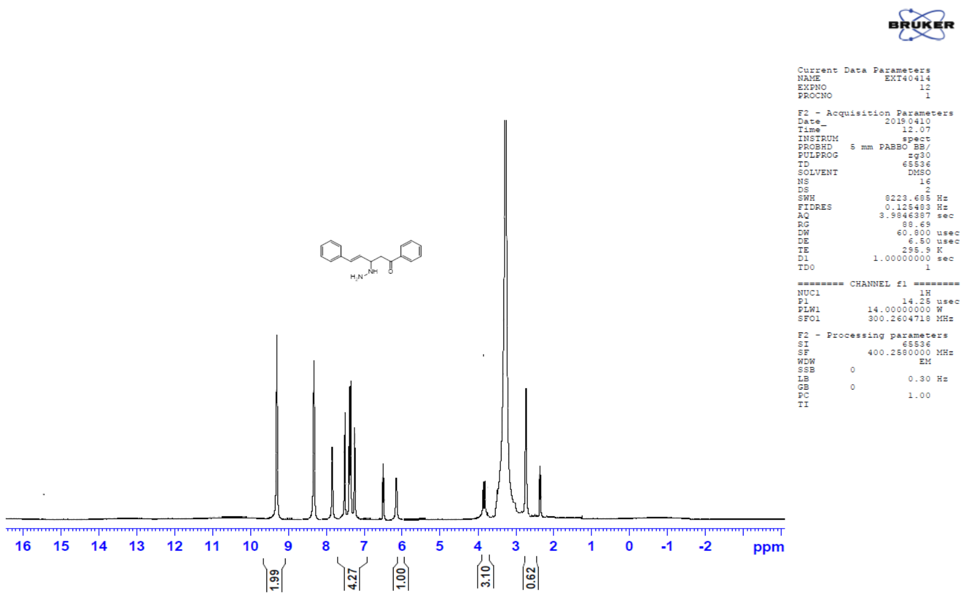

^{1}H NMR of the compound 1a

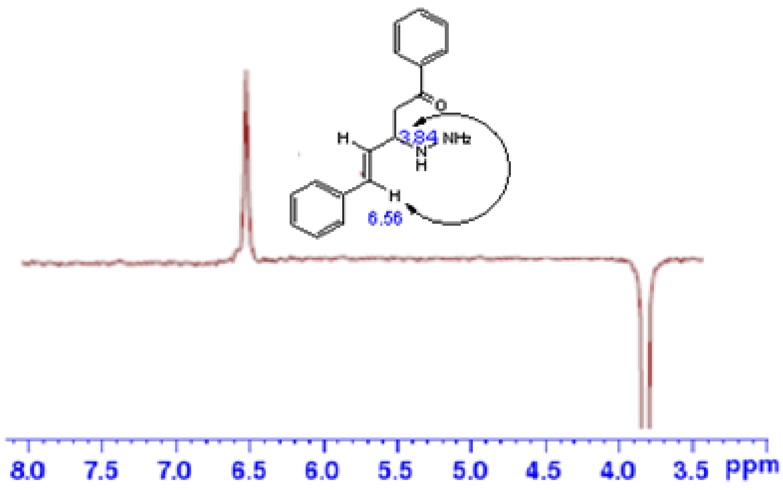

NOE Data for compound 1a

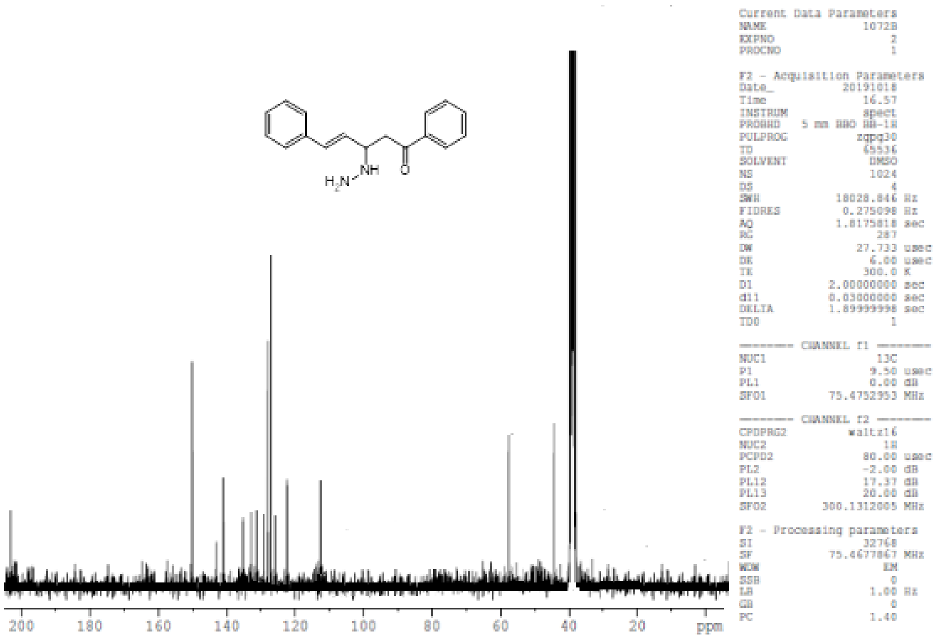

^{13}C NMR of the compound 1a

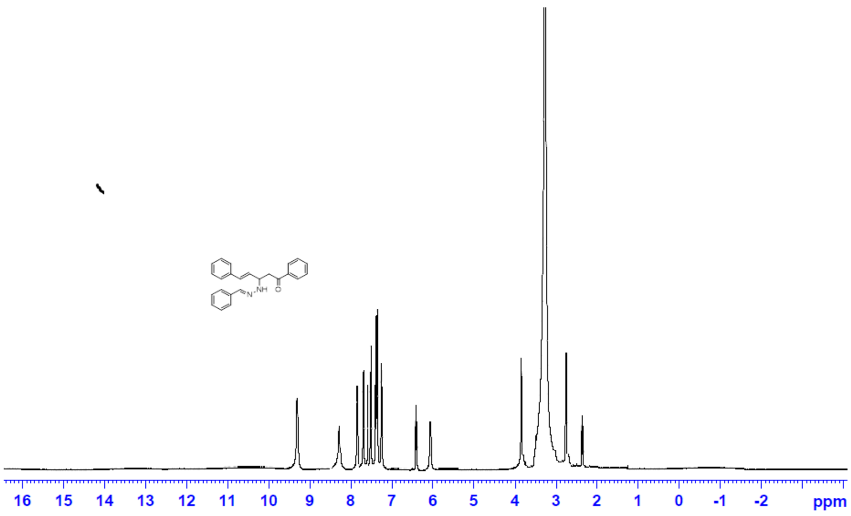

^{1}H NMR of the compound 1b

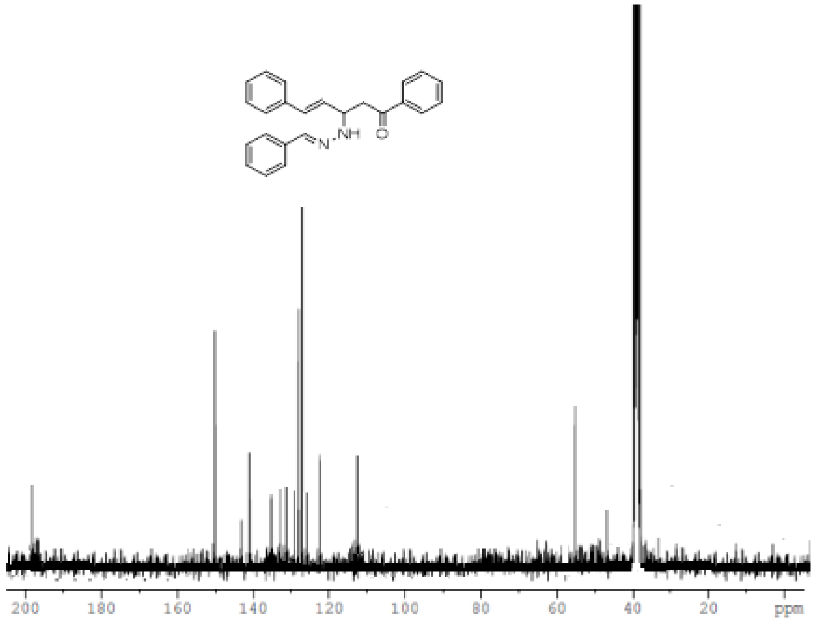

^{1}H NMR of the compound 1b

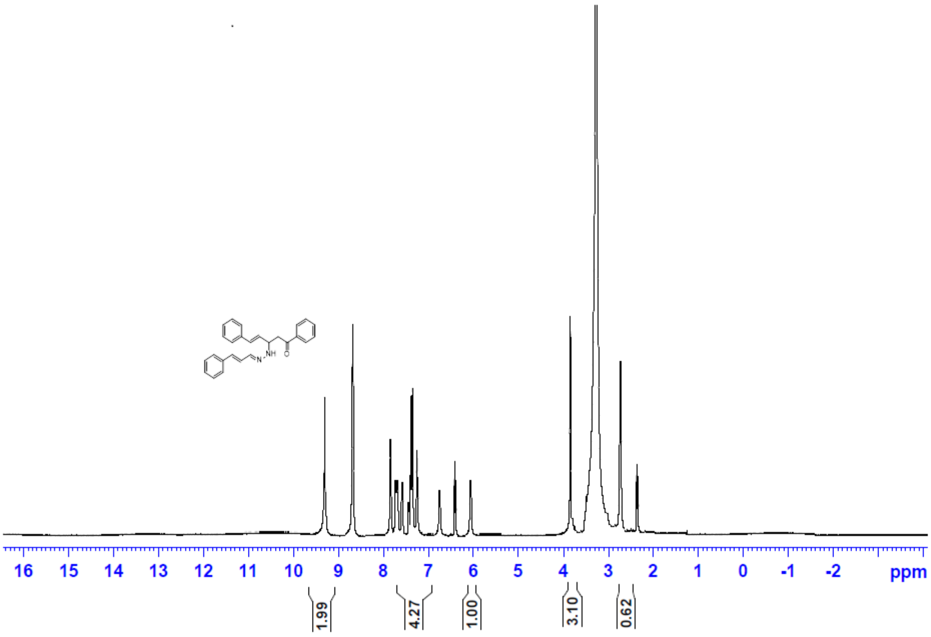

^{1}H NMR of the compound 1c

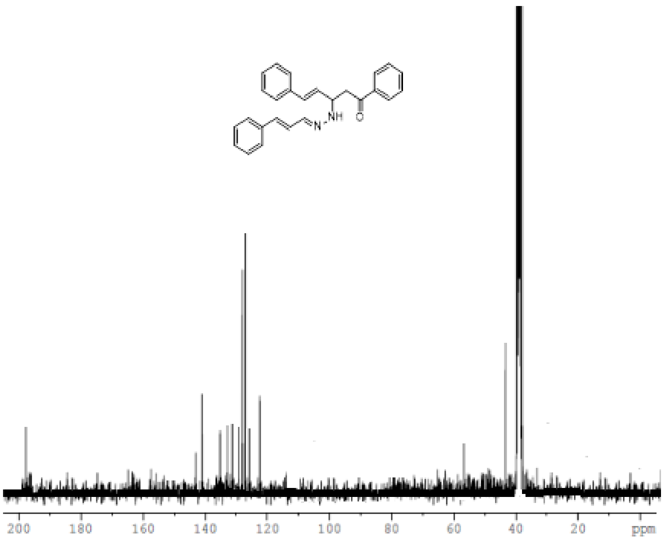

^{1}H NMR of the compound 1c

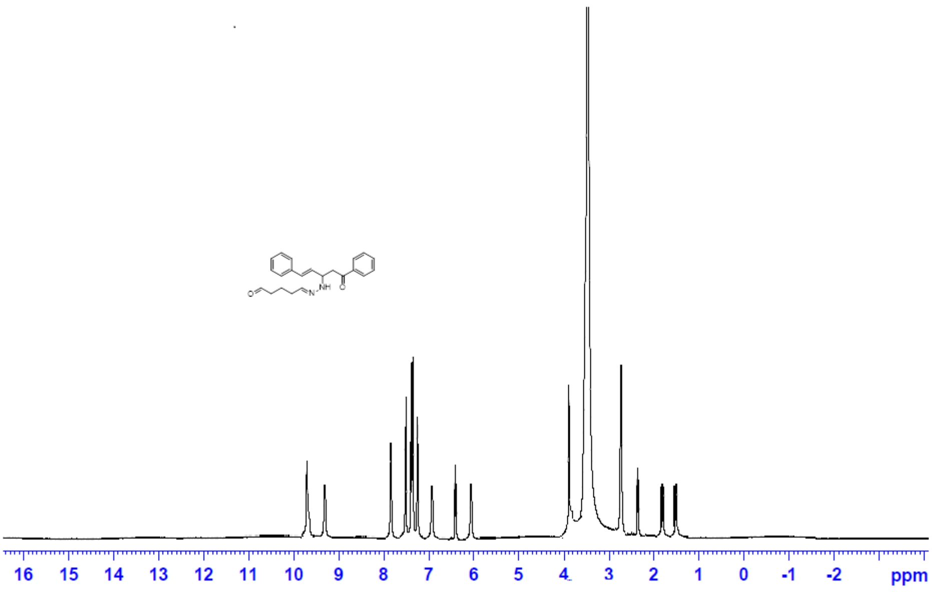

^{1}H NMR of the compound 1d

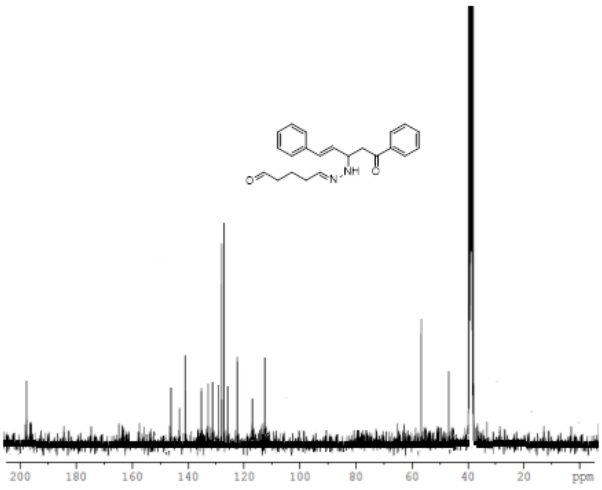

^{1}H NMR of the compound 1d

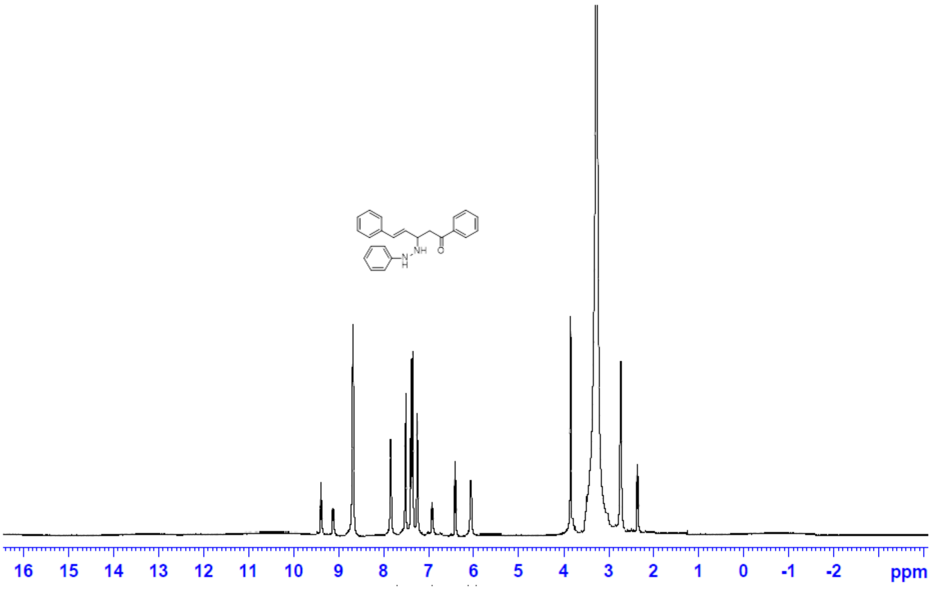

^{1}H NMR of the compound 1e

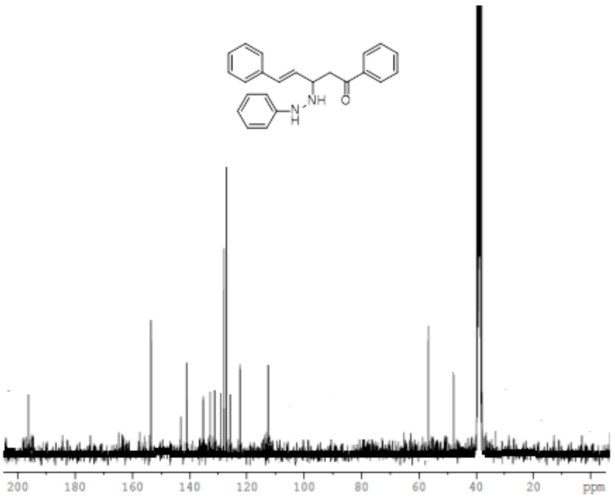

^{1}H NMR of the compound 1e

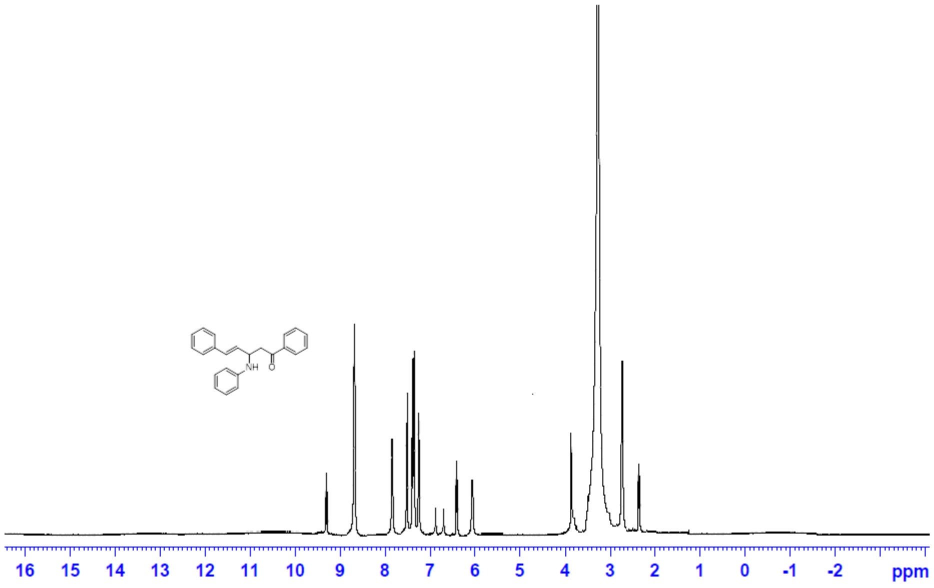

^{1}H NMR of the compound 1f

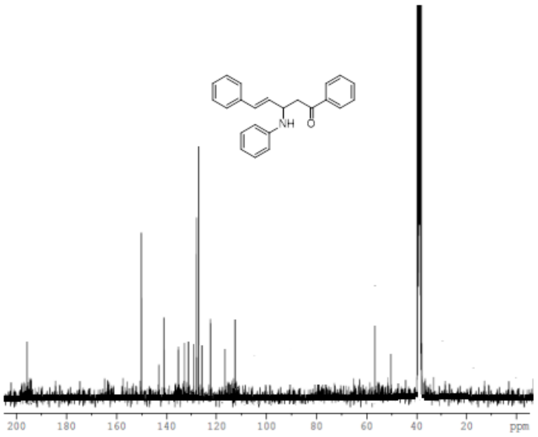

^{1}H NMR of the compound 1f

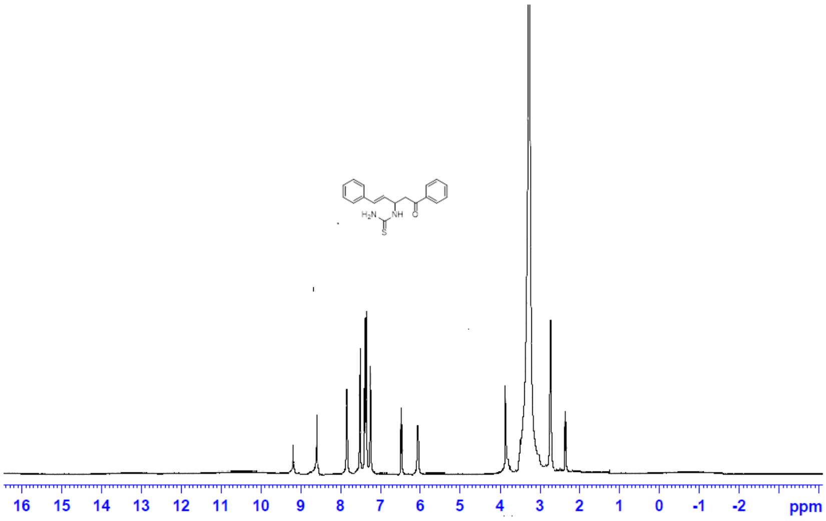

^{1}H NMR of the compound 1g

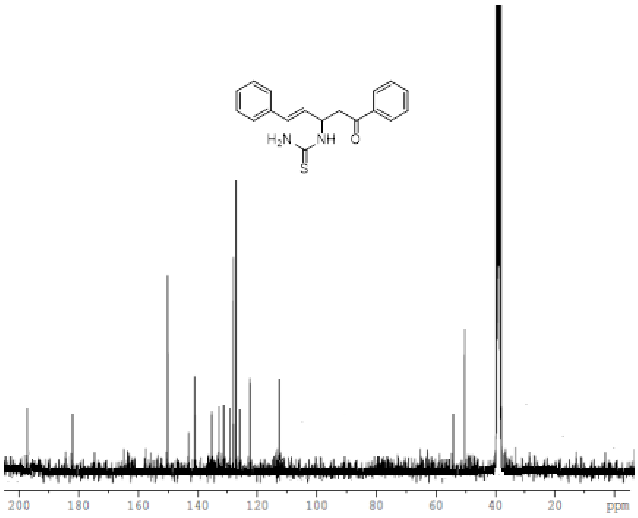

^{1}H NMR of the compound 1g

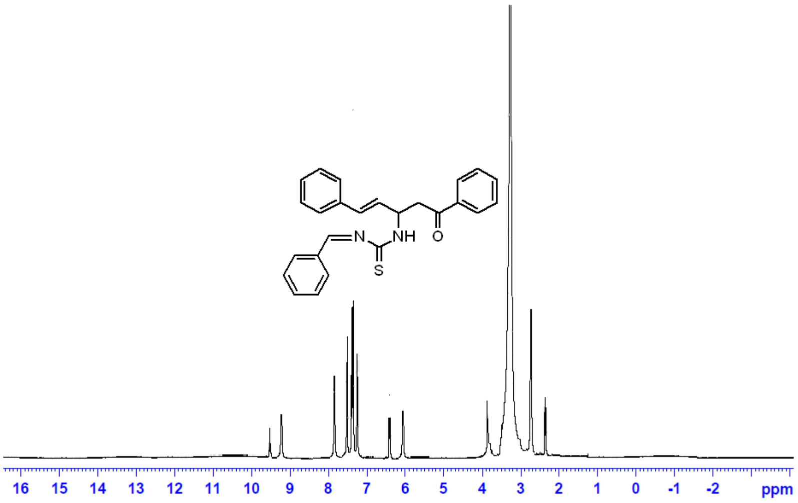

^{1}H NMR of the compound 1h

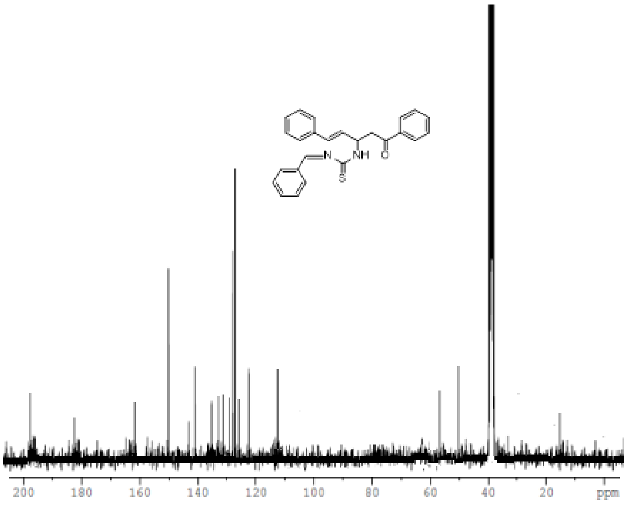

^{1}H NMR of the compound 1h

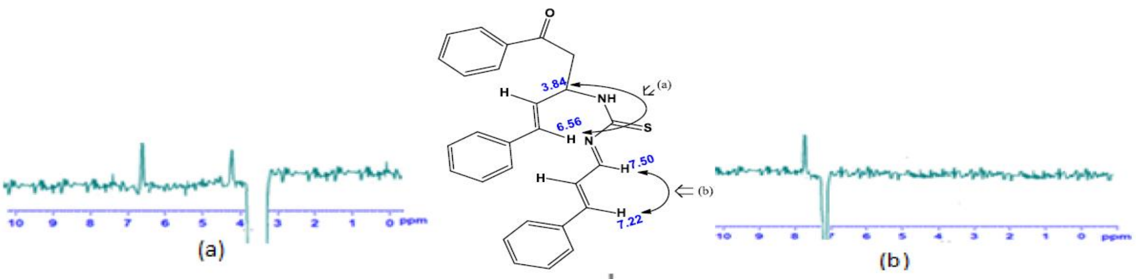

NOE Data for compound 1i

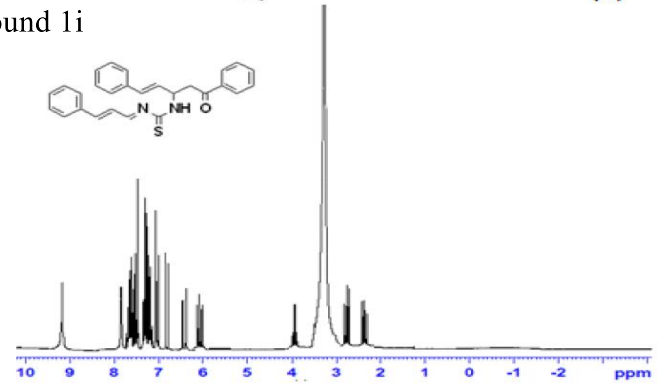

^{1}H NMR of the compound 1i

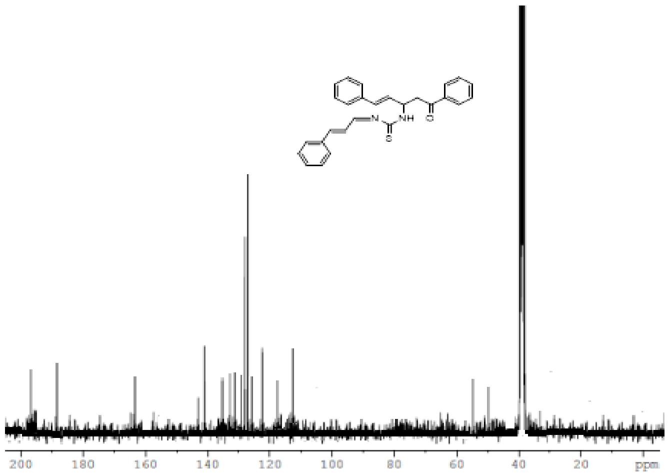

^{13}C NMR of the compound 1i

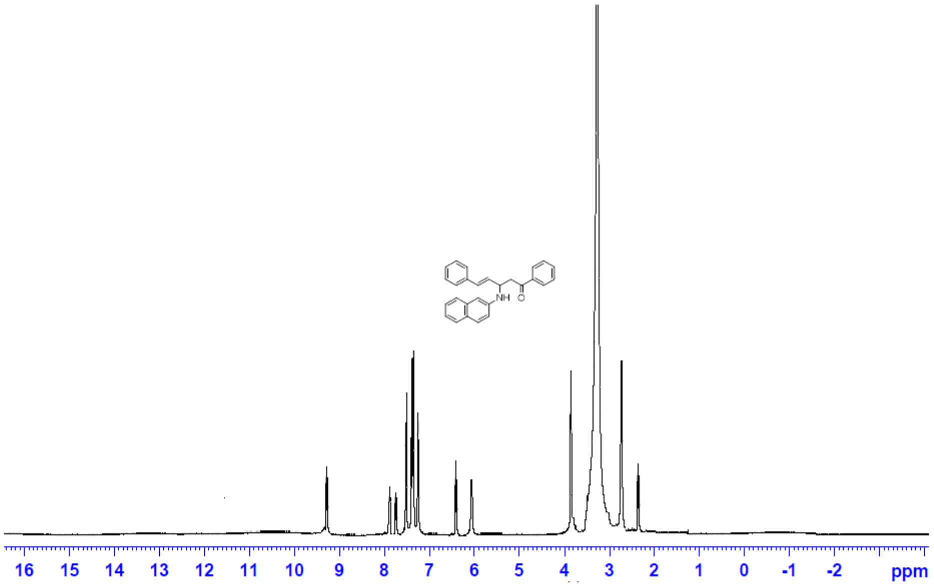

^{1}H NMR of the compound 1j

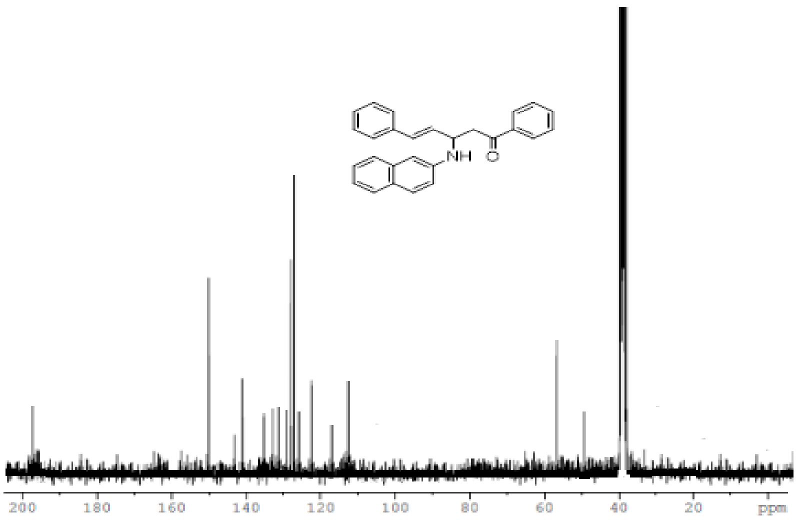

^{1}H NMR of the compound 1j

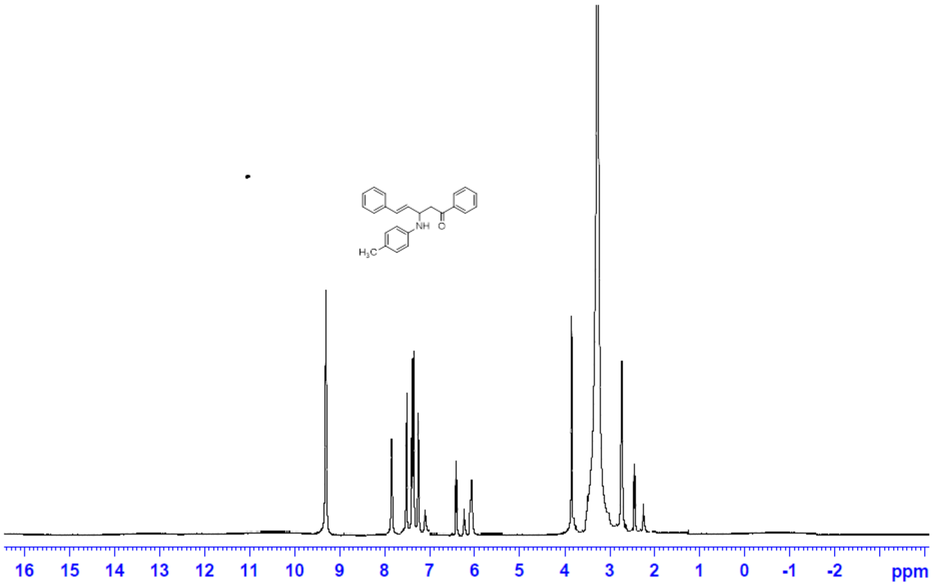

^{1}H NMR of the compound 1k

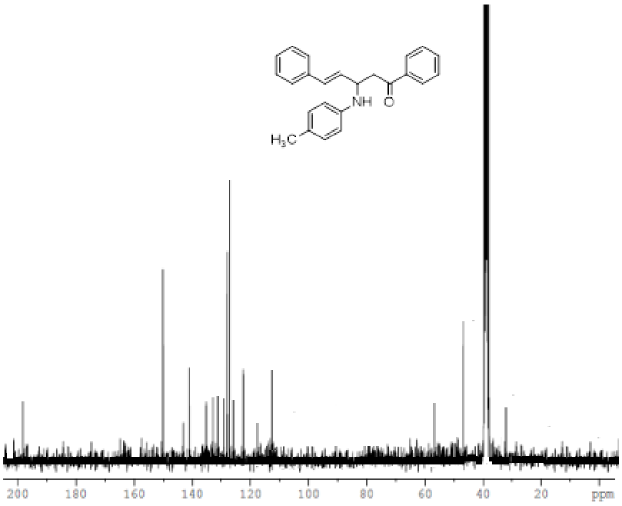

^{1}H NMR of the compound 1k

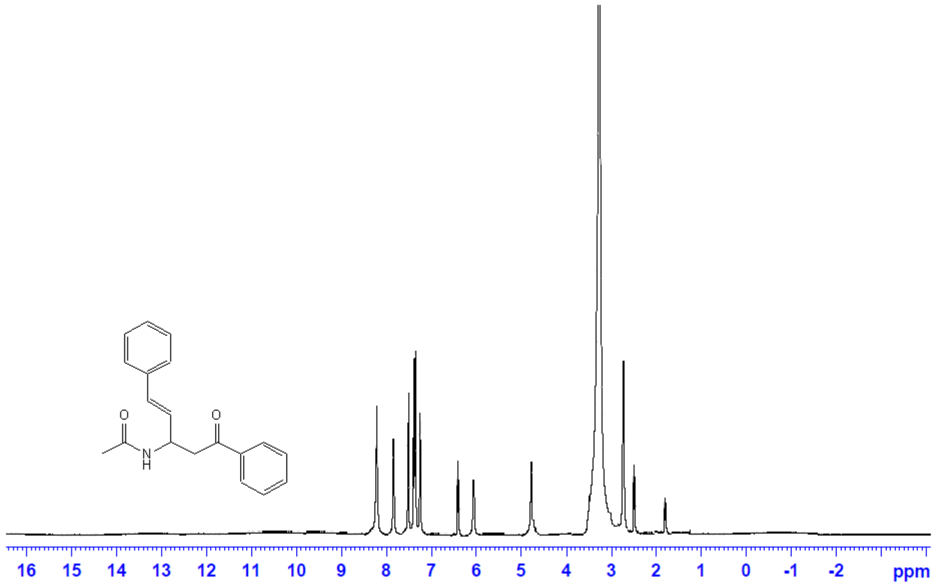

^{1}H NMR of the compound 1l

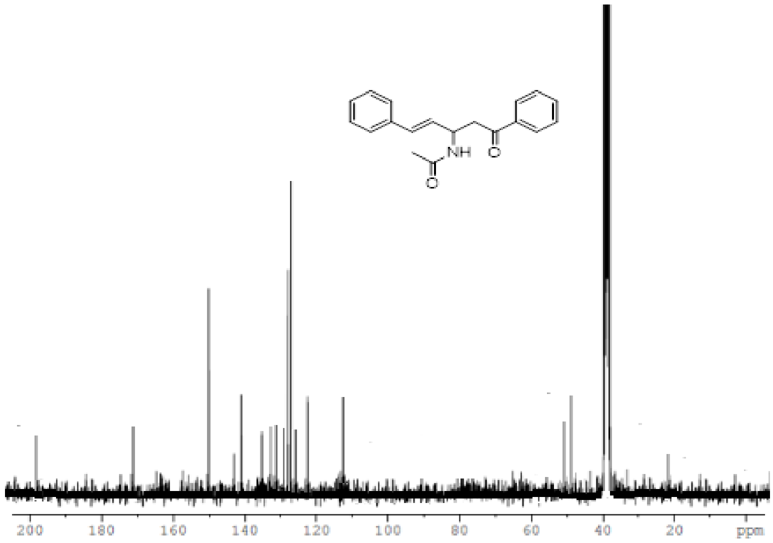

^{1}H NMR of the compound 1l

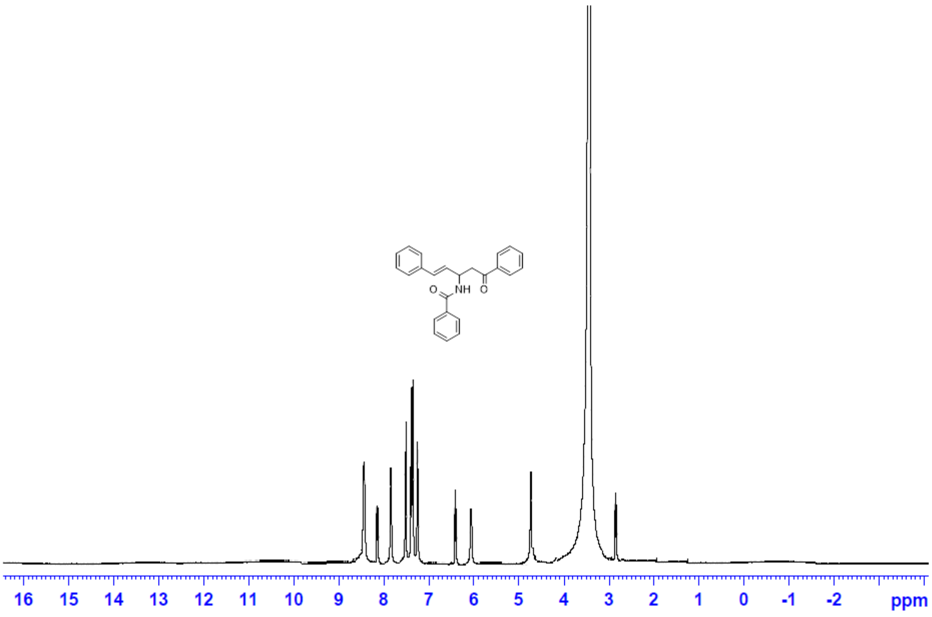

^{1}H NMR of the compound 1m

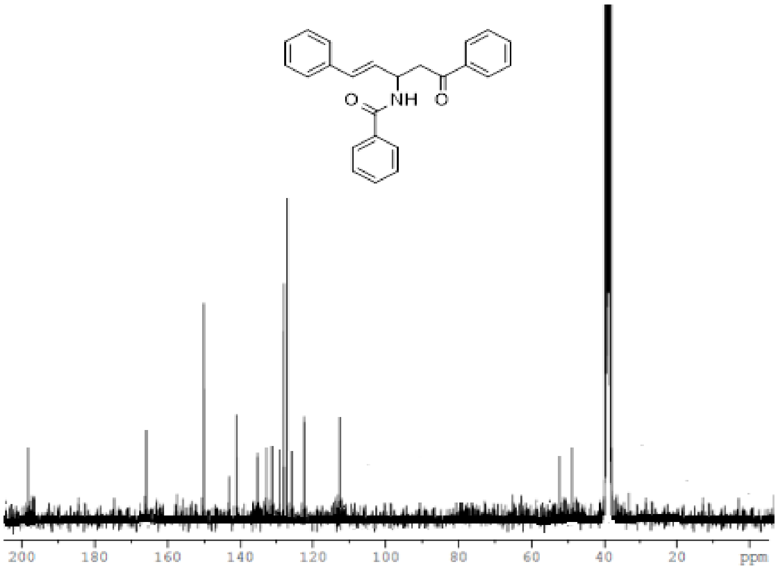

^{1}H NMR of the compound 1m

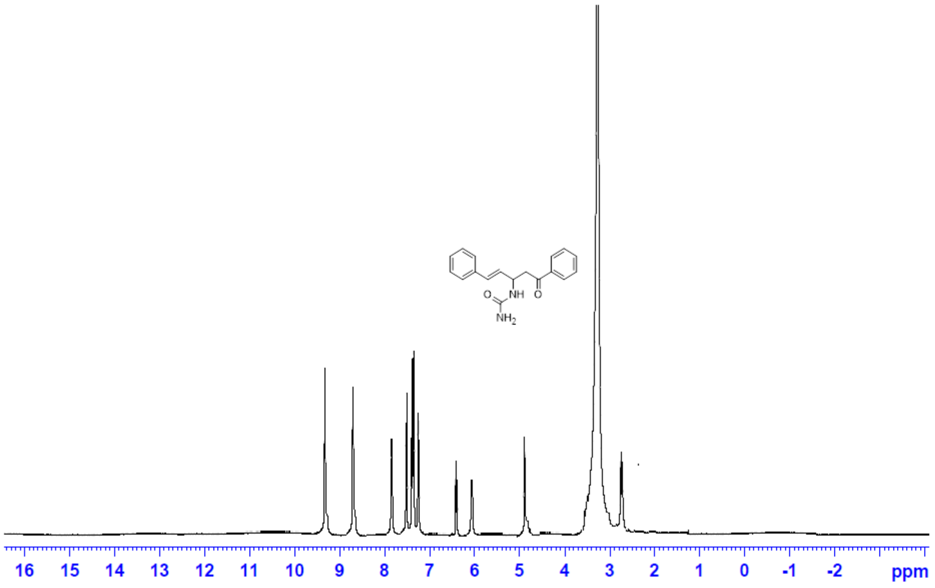

^{1}H NMR of the compound n

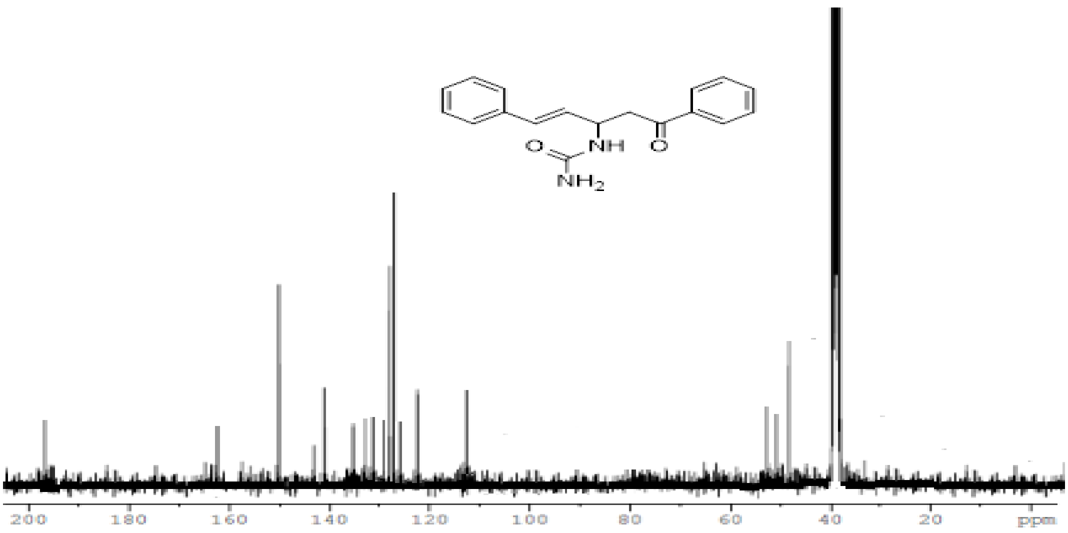

^{1}H NMR of the compound 1n

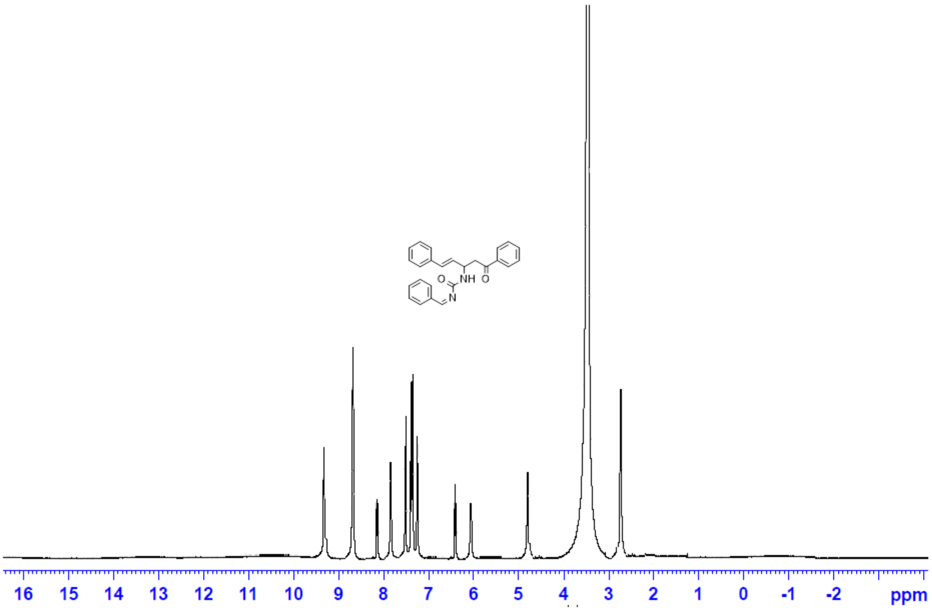

^{1}H NMR of the compound 1o

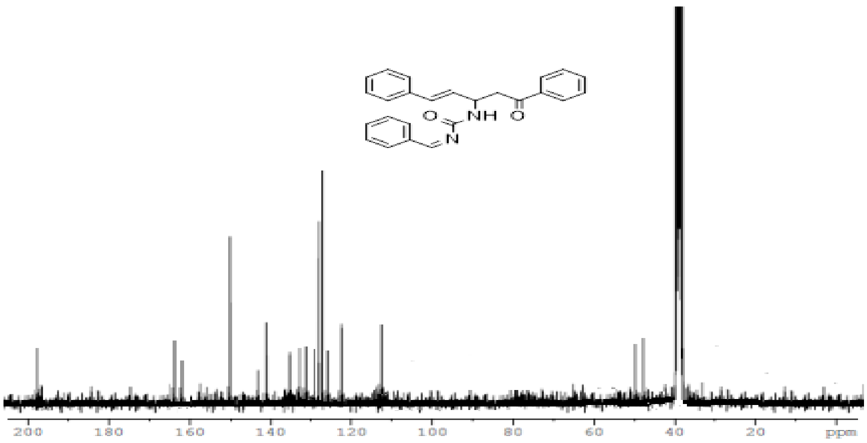

^{1}H NMR of the compound 1o

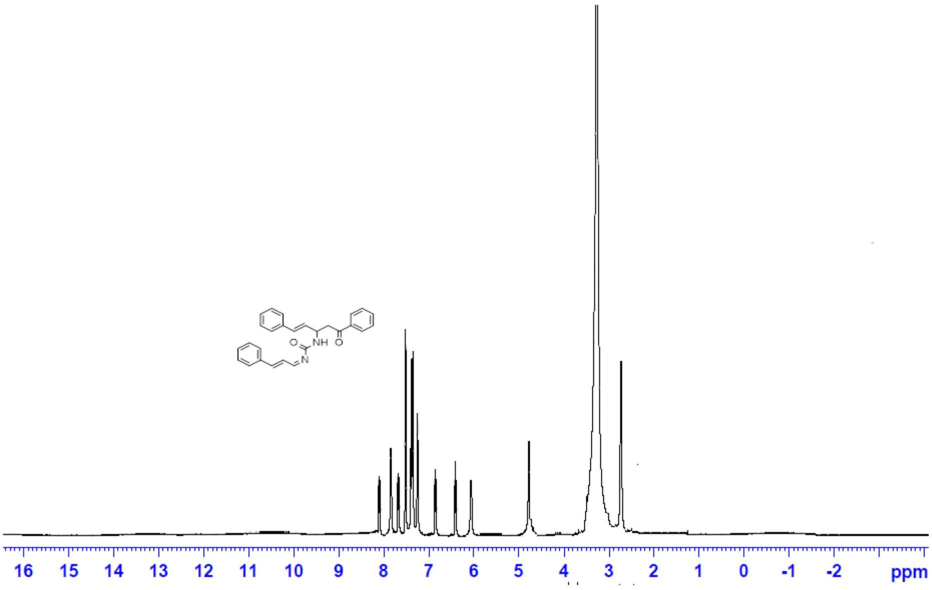

^{1}H NMR of the compound 1p

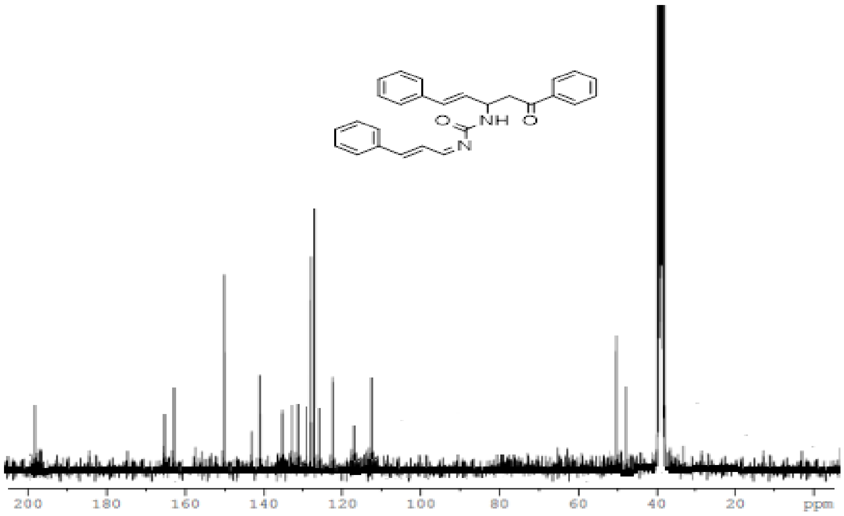

^{1}H NMR of the compound 1p

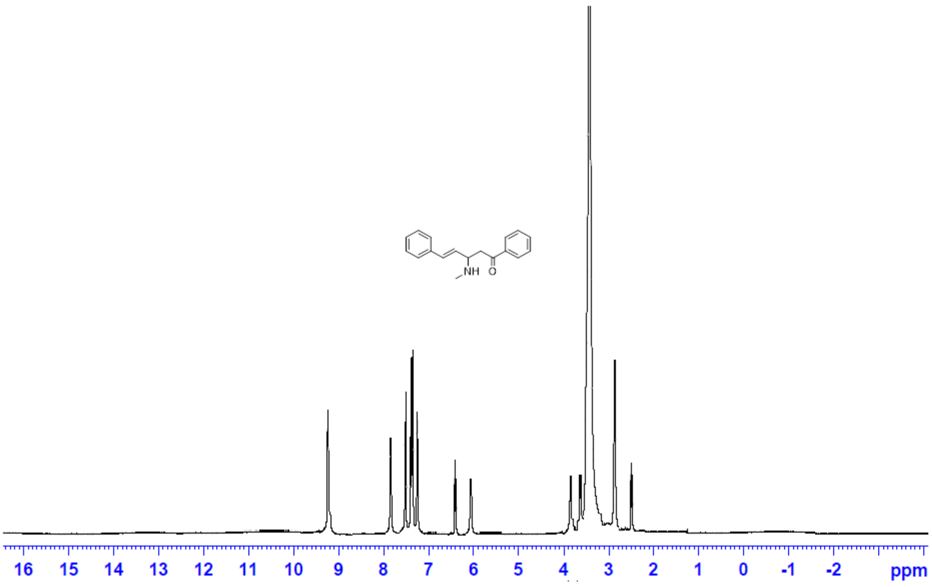

^{1}H NMR of the compound 1q

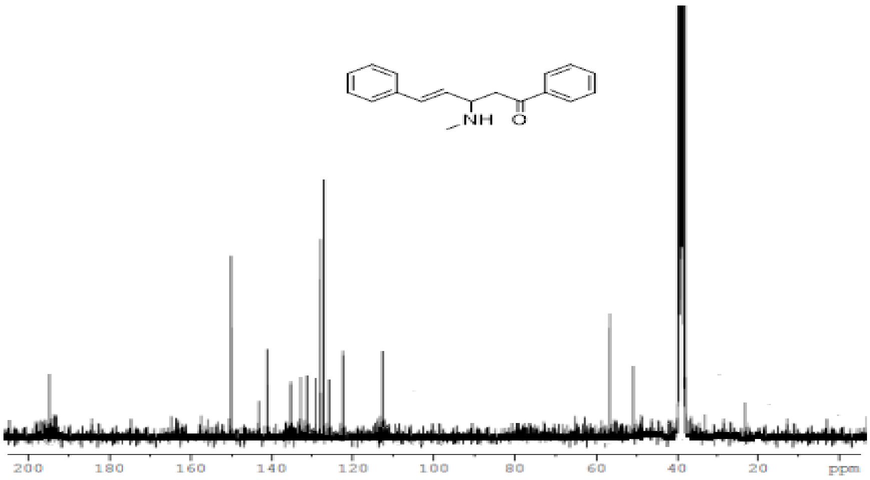

^{1}H NMR of the compound 1q

Table S1. Molecular docking interaction of compounds (**1a-1q**) and controls **Temephos**, **Permethrin**

<table>
<tr><th rowspan="2">Compounds</th><th colspan="4">Mosquito odorant-binding protein 3OGN</th></tr>
<tr><th>Binding affinity (kcal/mol)</th><th>No. of H-bonds</th><th>H-bonding residues</th><th>Interaction</th></tr>
<tr><td>1a</td><td>-9.0</td><td>2</td><td>His121, Phe123</td><td>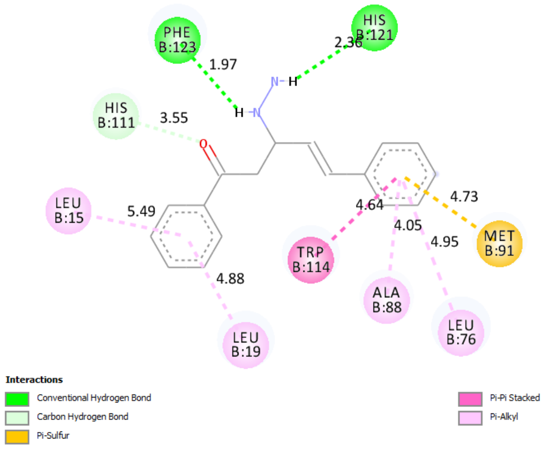
</td></tr>
<tr><td>1b</td><td>-9.7</td><td>0</td><td>-</td><td>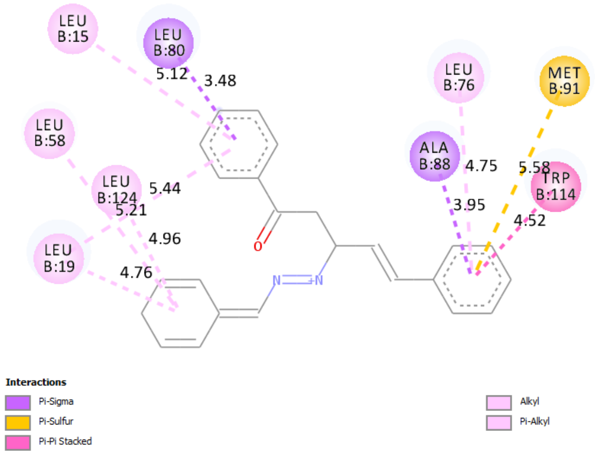
</td></tr>
<tr><td>1c</td><td>-9.0</td><td>0</td><td>-</td><td>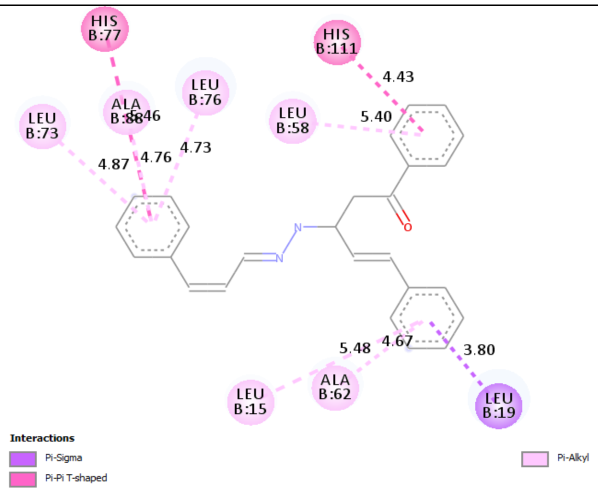
</td></tr>
</table>

**1d**	-8.8	0	-	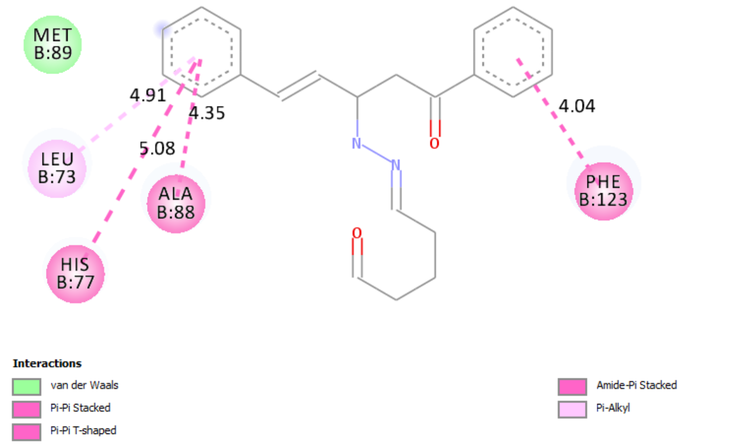
**1e**	-9.7	1	Phe123	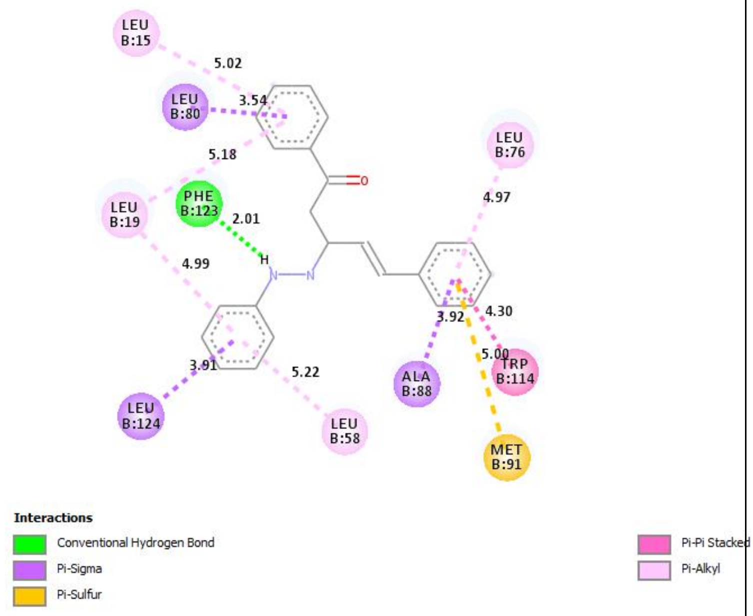
**1f**	-9.6	0	-	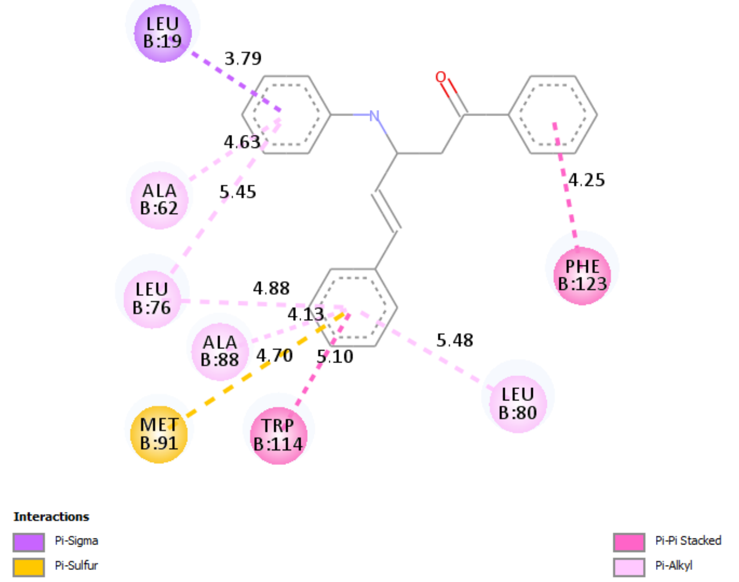

1g	-8.3	0	-	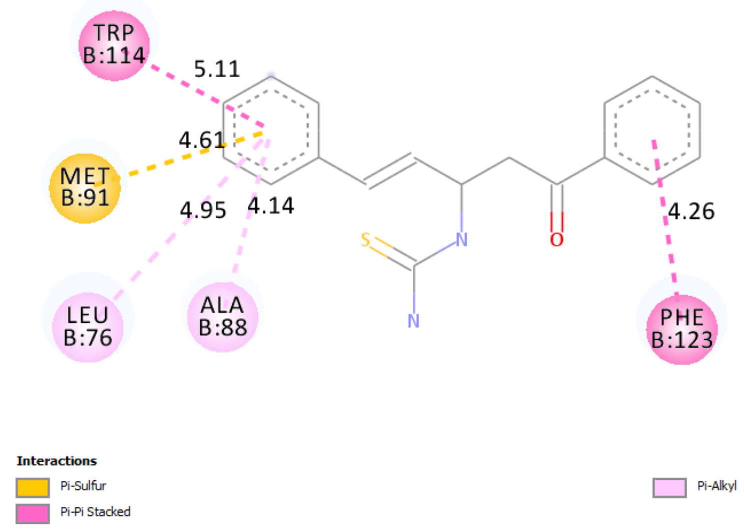
1h	-9.3	0	-	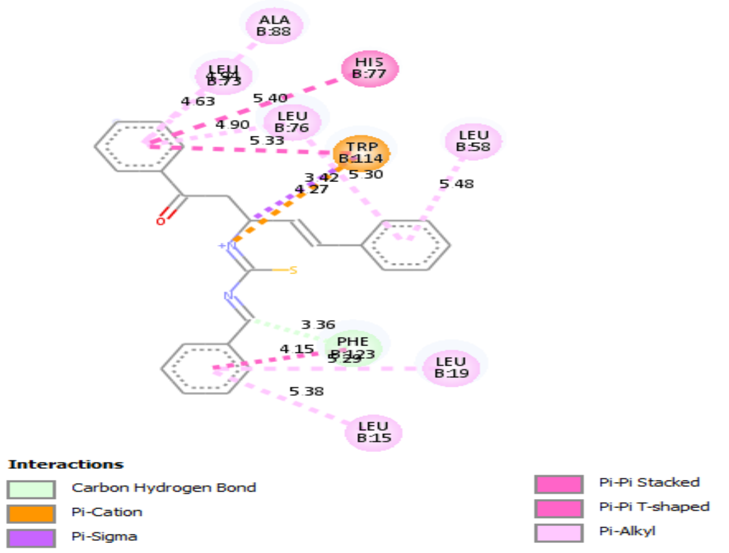
1i	-10.0	0	-	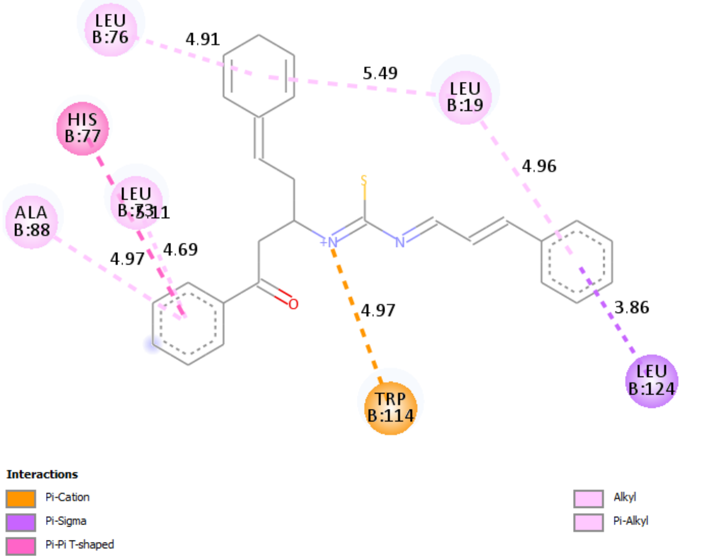

**1j**	-9.8	0	-	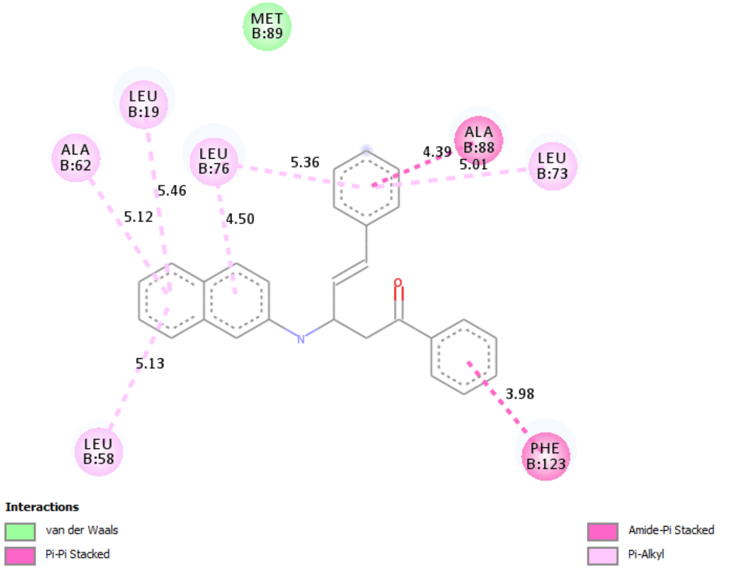
**1k**	-9.8	0	-	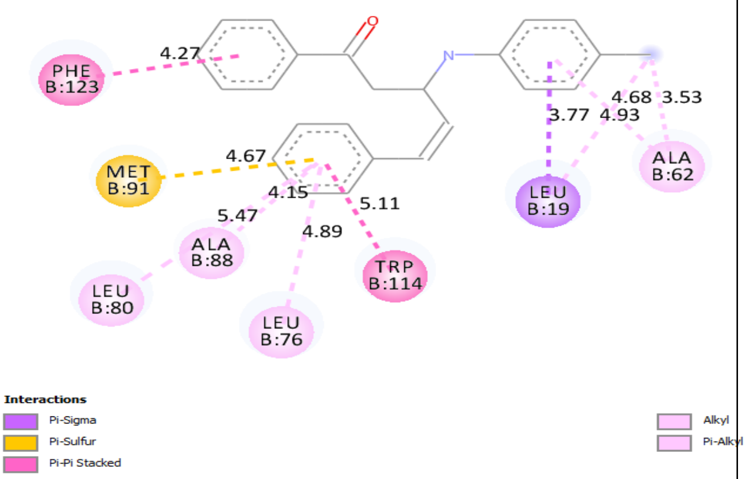
**1l**	-8.9	0	-	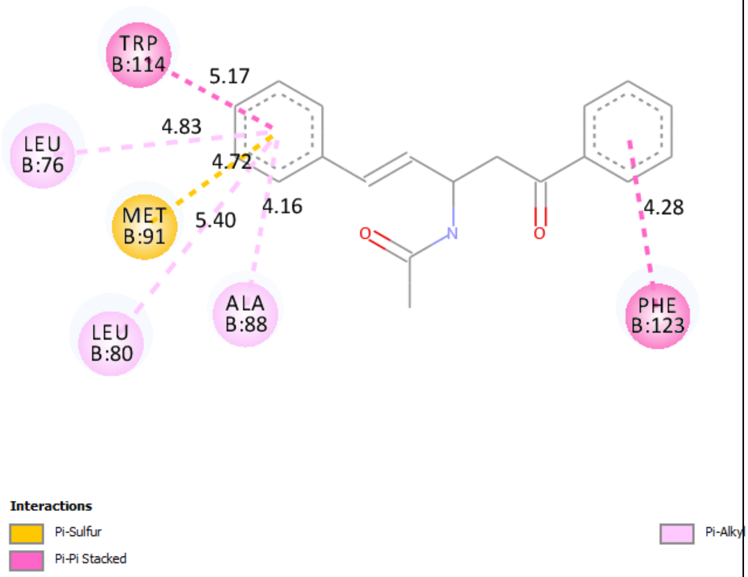

**1m**	-9.8	0	-	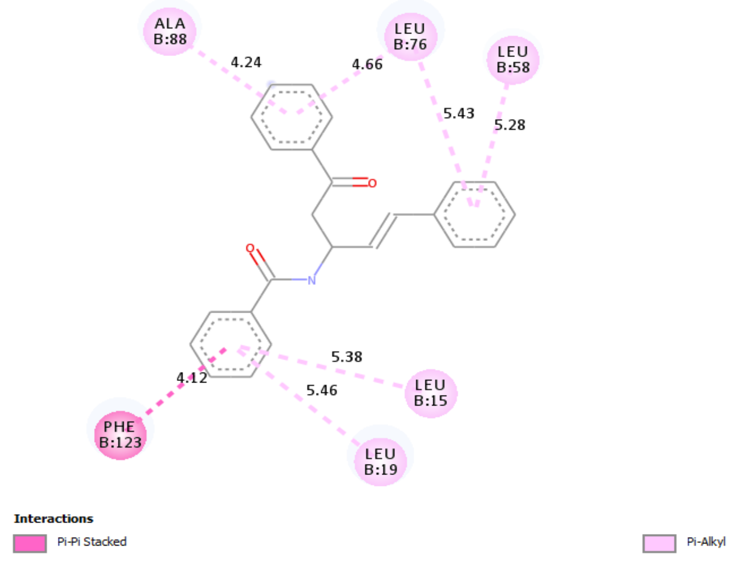
**1n**	-8.8	0	-	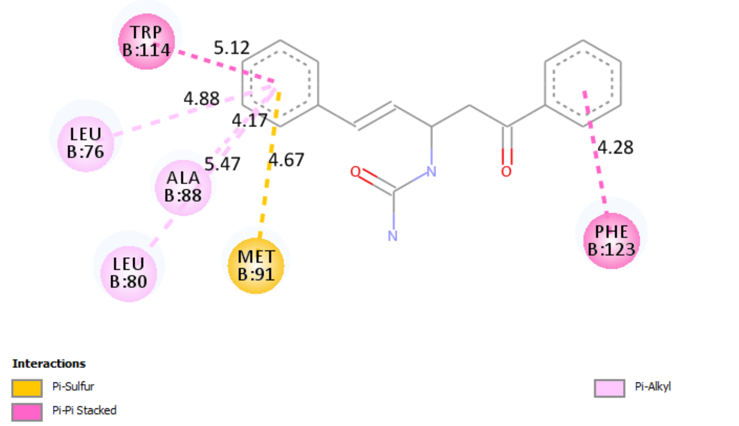
**1o**	-9.5	0	-	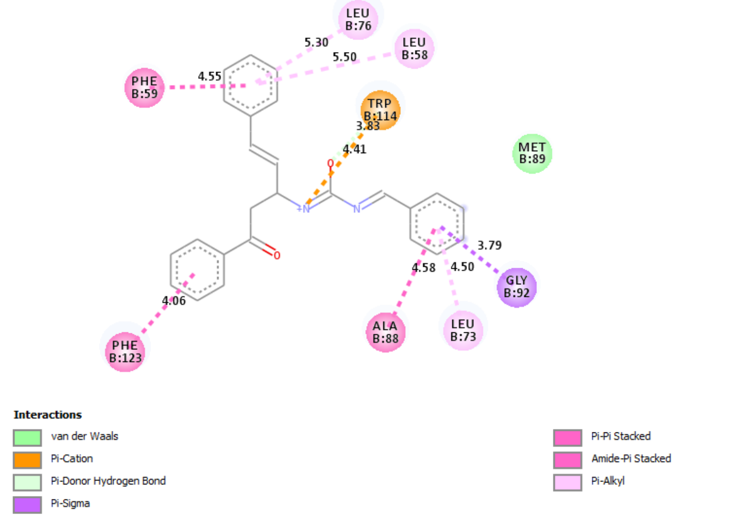

**1p**	-9.2	0	-	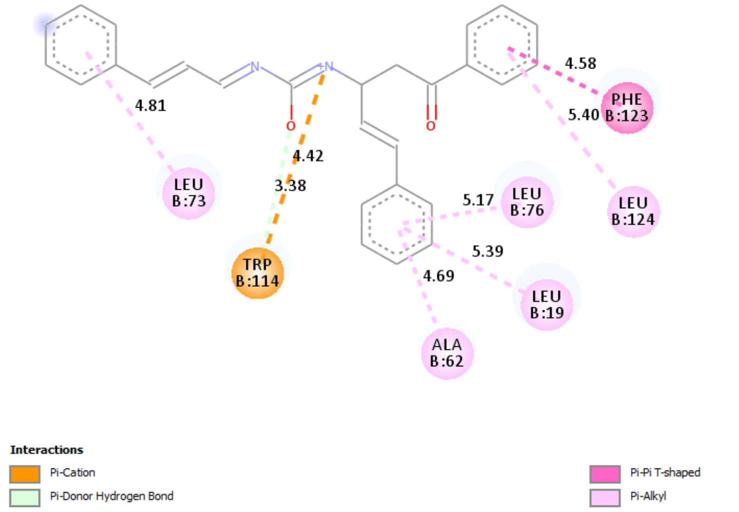
**1q**	-8.3	0	-	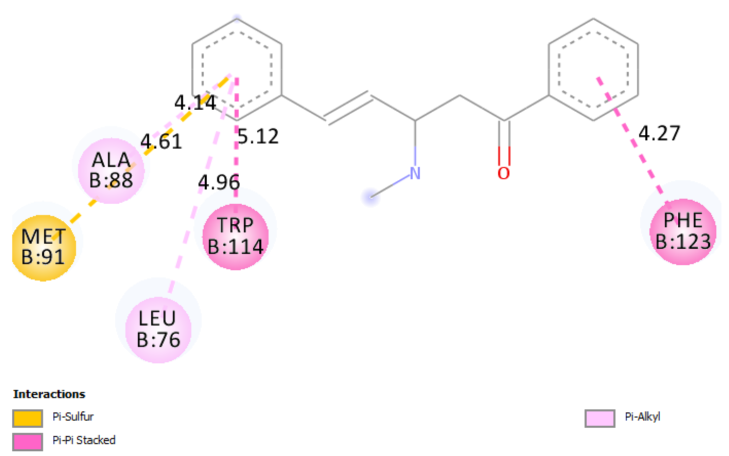
**Temephos**	-7.6	3	Ser79, Ala88	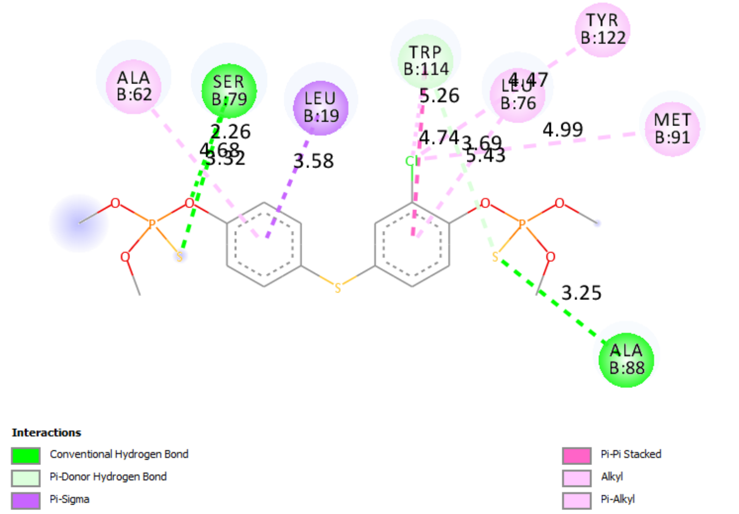

**Permethrin**	-9.7	0	-	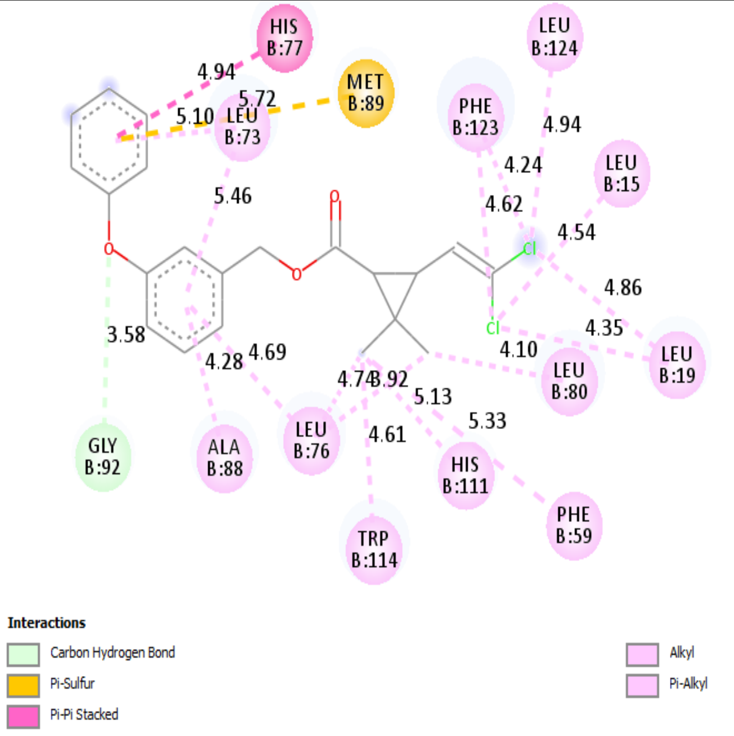
